# Complement inhibitor CSMD1 modulates epidermal growth factor receptor oncogenic signaling and sensitizes breast cancer cells to chemotherapy

**DOI:** 10.1186/s13046-021-02042-1

**Published:** 2021-08-17

**Authors:** Chrysostomi Gialeli, Emre Can Tuysuz, Johan Staaf, Safia Guleed, Veronika Paciorek, Matthias Mörgelin, Konstantinos S. Papadakos, Anna M. Blom

**Affiliations:** 1 Department of Translational Medicine, Lund University, Malmö, Sweden; 2 Experimental Cardiovascular Research Group, Department of Clinical Sciences, Lund University, Malmö, Sweden; 3 Division of Oncology, Department of Clinical Sciences Lund, Lund University, Medicon Village, Lund, Sweden; 4 Colzyx AB, Lund, Sweden

**Keywords:** CSMD1, EGFR trafficking, Chemosensitivity, Breast cancer

## Abstract

**Background:**

Human CUB and Sushi multiple domains 1 (CSMD1) is a large membrane-bound tumor suppressor in breast cancer. The current study aimed to elucidate the molecular mechanism underlying the effect of CSMD1 in highly invasive triple negative breast cancer (TNBC).

**Methods:**

We examined the antitumor action of CSMD1 in three TNBC cell lines overexpressing CSMD1, MDA-MB-231, BT-20 and MDA-MB-486, in vitro using scanning electron microscopy, proteome array, qRT-PCR, immunoblotting, proximity ligation assay, ELISA, co-immunoprecipitation, immunofluorescence, tumorsphere formation assays and flow cytometric analysis. The mRNA expression pattern and clinical relevance of *CSMD1* were evaluated in 3520 breast cancers from a modern population-based cohort.

**Results:**

CSMD1-expressing cells had distinct morphology, with reduced deposition of extracellular matrix components. We found altered expression of several cancer-related molecules, as well as diminished expression of signaling receptors including Epidermal Growth Factor Receptor (EGFR), in CSMD1-expressing cells compared to control cells. A direct interaction of CSMD1 and EGFR was identified, with the EGF-EGFR induced signaling cascade impeded in the presence of CSMD1. Accordingly, we detected increased  ubiquitination levels of EGFR upon activation in CSMD1-expressing cells, as well as increased degradation kinetics and chemosensitivity. Accordingly, CSMD1 expression rendered tumorspheres pretreated with gefitinib more sensitive to chemotherapy. In addition, higher mRNA levels of *CSMD1* tend to be associated with better outcome of triple negative breast cancer patients treated with chemotherapy.

**Conclusions:**

Our results indicate that CSMD1 cross-talks with the EGFR endosomal trafficking cascade in a way that renders highly invasive breast cancer cells sensitive to chemotherapy. Our study unravels one possible underlying molecular mechanism of CSMD1 tumor suppressor function and may provide novel avenues for design of better treatment.

**Supplementary Information:**

The online version contains supplementary material available at 10.1186/s13046-021-02042-1.

## Background

Human CUB and Sushi Multiple Domains 1 (CSMD1) is a large (390 kDa) transmembrane complement inhibitor, highly expressed in testis and brain, but also present in lung, colon, thyroid gland, breast, and pancreas [1–3]. The large, extracellular portion of the protein consists of 14 N-terminal CUB domains, interspaced by single complement control protein (CCP) domains and a subsequent tandem array of 15 CCP domains. The highly conserved C-terminal part is composed of a single transmembrane region followed by a short cytoplasmic tail of 56 amino acids accommodating several predicted phosphorylation sites on serine, threonine and tyrosine residues. CSMD1 is therefore a potential receptor/co-receptor with downstream signaling [4, 5] but no physiological ligands triggering its signaling have to date been identified.

The *CSMD1* chromosomal locus spans over 2 million base pairs at the short arm of chromosome 8 (8p23.1) [4, 6]. Using genome-wide association studies, CSMD1 has been associated with several pathological processes, from neurodegenerative and psychiatric disorders to infertility and cancer [7–9]. For several years, CSMD1 has been proposed to act as a tumor suppressor since allelic loss, mutations, and methylations in its genomic region have been reported in several malignancies, including breast cancer [6, 10–15]. In addition, a decrease in *CSMD1* expression has been linked to poor prognosis and shorter survival of cancer patients [14, 16–18]. However, there is only a handful of reports experimentally confirming the tumor suppressing properties of CSMD1 [18–23]. Thus, abrogation of CSMD1 expression in normal mammary human cells disrupts normal breast cell function and transformation, rendering the cells highly proliferative, migratory and invasive [22]. Forced expression of CSMD1 in human BT-20 and MDA-MB-231 triple negative breast cancer (TNBC) cells significantly inhibited their migration, adhesion and invasion, while stable silencing of CSMD1 expression in hormone-dependent T47D cells enhanced their migratory, adherent and clonogenic abilities. Moreover, expression of CSMD1 in the highly invasive MDA-MB-231 cells diminished their overall signaling potential and stem cell-like properties. Further, in a xenograft model, expression of CSMD1 entirely blocked the ability of MDA-MB-231 cells to metastasize in vivo, likely via inhibiting local invasion, but not extravasation into distant tissues [18]. However, the molecular mechanisms responsible for the suppressor effects of CSMD1 are unknown, and the current study therefore aimed to identify them.

## Methods

### Cell lines, culture conditions and mammary tissues

The human breast cancer cell lines MDA-MB-231, BT-20 and MDA-MB-468 were purchased directly from ATCC and all the experiments were performed on low passage cultures. Cells stably transfected to express CSMD1 were cultured with addition of 3 μg/ml puromycin (Invitrogen). The generation of the stable transfectants has been described previously [18]. All cells were maintained in DMEM (HyClone) supplemented with 10% fetal bovine serum (FBS) and penicillin/streptomycin. All cells were tested for *Mycoplasma* contamination monthly. Throughout the manuscript CSMD1-expressing cells are denoted as CSMD1, while those containing the control empty-GFP vector were coded as CTRL. Cells were starved in serum-free medium for 2 h prior to all stimulation experiments. All stimulations were carried out in starvation medium for the indicated times.

### Scanning electron microscopy

Cells were seeded on plastic cover slips and after a 24 h culture period were further processed as described previously [24].

### Proteome profiling

A Proteome Profiler™ Human XL Oncology Array kit (R&D, ARY026) was used to determine the relative levels of 84 human-cancer-related proteins according to manufacturer's instructions. CTRL and CSMD1-expressing MDA-MB-231 breast cancer cells were serum starved for 30 min to synchronize them and then lysed in the provided lysis buffer (300 μg protein/array). Cell culture supernatants were collected in Optimem serum free medium (Gibco) for 48 h and subsequently concentrated five times using Amicon filters (Millipore, 2 kDa cutoff). Densities of individual dots corresponding to each protein were measured by the Image J software to compare CTRL and CSMD1-expressing cells.

### Real time PCR

RNA was purified using RNAeasy Plus Mini kits (Qiagen) from tumors formed in vivo after orthotopic injection of MDA-MB-231 CTRL and CSMD1 cells into the mammary fat pad of 8 weeks old SCID (CB-17/Icr-Prkdcscid/Rj) mice as described [18], or from MDA-MB-231 CTRL and CSMD1 cells grown in vitro*.* Next, mRNA was reverse transcribed to cDNA with SuperScript III Reverse Transcriptase and gene expression was quantified using TaqMan Gene Expression Assays. mRNA expression levels were calculated using the ΔCT method after normalization with the geometric mean of the three housekeeping genes; *cyclophilin A* (Hs99999904_m1), *TATA box binding protein* (Hs0042761_m1) and *hypoxanthine phosphoribosyltransferase* 1 (Hs99999909_m1). Genes assayed and their corresponding TaqMan Gene Expression Assays from ThermoFisher were: *EGFR* (Hs01076090m1), *CTSS* (Hs00175407m1), *EGF* (Hs01099990m1), *TGFα* (Hs00608187), and *AREG* (Hs00950669m1).

### Flow cytometry

Cells were seeded in 6-well plates and once 70% confluency was reached, detached with Versene (EDTA based buffer, Invitrogen), washed with FACS buffer (10 mM HEPES, 140 mM NaCl, 5 mM KCl, 1 mM MgCl_2_, 2 mM CaCl_2_, 0.02% NaN_3_), and incubated for 1 h with the appropriate antibody in FACS buffer at room temperature. After washing with FACS buffer cells were analyzed using Cytoflex flow cytometer (Beckman) and FlowJo software (BD). For discrimination between live, apoptotic, or necrotic cells, cells were stained with Annexin V-APC (Immunotools) and live/dead cell discrimination reagent Zombie Aqua (Biolegend) in FACS buffer for 30 min, and then analyzed as above.

### Immunoprecipitation & Immunodetection of proteins

Total protein lysates were obtained with RIPA buffer (150 mM NaCl, 10 mM Tris–HCl [pH 7.2], 0.1% SDS, 1% Triton X-100) or NP40 buffer (150 mM NaCl, 50 mM Tris–HCl [pH 7.5], 1% NP40) containing a protease and phosphatase inhibitor cocktail (Thermo Fisher Scientific). When ubiquitination experiments were performed, lysates were also supplemented with DUBs inhibitor N-Ethylmaleimide (NEM). Protein concentrations in lysates were estimated with Pierce BCA Protein Assay Kit, equal amounts were separated by SDS-PAGE, and transferred onto PVDF membranes with a Trans-Blot Turbo Transfer Pack (Bio-Rad). For immunoprecipitation, 1 mg of total protein was used with 50 μl of Protein G or Protein A Dynabeads pre-coated with 5 μg of capturing antibody. After overnight incubation, beads were extensively washed with NP40 buffer, and immunoprecipitated proteins were eluted with 30 μl of Laemmli buffer. Primary and secondary antibodies used are listed in Table S1.

### Transfection

Transfection of DNA constructs was performed using Lipofectamine 2000 (Invitrogen) following the manufacturer’s instructions. 1 μg of DNA was used for each transfection. pcDNA6A-EGFR ECD (1–644) (Addgene plasmid #42,666; http://n2t.net/addgene:42666; RRID:Addgene_42666) and pcDNA6A-EGFR ICD (645–1186) (Addgene plasmid # 42,667; http://n2t.net/addgene:42667; RRID:Addgene_42667) were a gift from Dr Mien-Chie Hung [25].

### Immunofluorescent & dual link assay

Imaging chamber slides from Ibidi were coated with Poly-D-lysine (Sigma), before cells seeding. The following day, cells were fixed with 4% formaldehyde (Merck) and permeabilized with 0.1% Triton X-100 (Merck). Proximity ligation assays was performed following the manufacturer's protocol for the Dual link kit (Sigma) using the appropriate antibodies (Table S1). For the immunofluorescent studies, cells were fixed as described above, blocked with 3% BSA in PBS for 1 h at room temperature, and incubated with primary antibodies overnight at 4 °C, followed by 1 h incubation with the corresponding Alexa-conjugated secondary antibodies at room temperature. Cells were washed and mounted using Prolong Glass medium (ThermoFisher Scientific) and visualized with ZEIS LSM800 confocal microscope. Nuclei were stained with DAPI or SYTOX Orange. Corresponding IgG controls were used as negative controls for each antibody staining. Co-localization quantification was performed using Manders’ coefficient in Coloc2 plugin of FIJI software. The Manders M1 coefficient was expressed as a percentage (e.g. M1 = 0.2 was expressed as 20%) to show the fraction of intensities in channel 1 that is colocalized with intensities in channel 2.

### EGFR degradation

Cells were seeded and incubated overnight. Next day, cells were pre-treated with 100 μM of cycloheximide (Sigma Aldrich) for 2 h and stimulated with EGF (Sigma Aldrich) in the presence of cycloheximide for indicated time points. Cells incubated with only cycloheximide were used to determine the initial amount of EGFR for each clone. NH_4_Cl (10 nM) and MG132 (5 μM) were used to evaluate lysosome and proteasome contributions in EGFR degradation, respectively. EGFR recovery was assessed by calculating the ratio [Cyclo + NH_4_Cl (or MG132) + EGF] / [Cyclo + EGF].

### Detection of EGFR Dimers

EGFR dimers were detected as described [26]. Briefly, cells were stimulated with EGF (25 ng/ml) and/or Gefitinib (GEF; 10 μM, Selleckchem) for 15 min at 37 °C. Following stimulation, cell surface proteins were cross-linked with 1.1 mM of bis[sulfosuccinimidyl] suberate (BS^3^, Thermo Scientific) for 30 min at room temperature. Cells were lysed and equal amounts of protein were separated by 3–8% Criterion™ XT Tris–Acetate Protein Gel (Biorad), transferred onto PVDF membranes with Trans-Blot Turbo Transfer Pack and probed for EGFR using specific antibodies.

### Tumorsphere formation assay

Cells at 1.5 × 10^5^ (for MDA-MB-231) or 1 × 10^5^ (for BT-20) per well were seeded onto AggreWell™400 plates (Stem Cell) and maintained in MammoCult™ Human Medium Kit (Stem Cell) according to manufacturer’s instructions to develop uniform tumorpheroids with consistent size and shape. Next day, medium was replaced with fresh medium containing 35 μM of GEF followed by 2 μM of doxorubicin or epirubicin treatment for two days (MDA-MB-231) or 8 μM of doxorubicin or epirubicin treatment for one day (BT-20).

### Gene expression analysis of breast cancer tumors

Gene expression profiles for 3520 primary breast cancer patients from the Swedish Cancerome Analysis Network – Breast (SCAN-B) study were obtained from the study by Vallon-Christersson et al*.,* as processed fragments per kilobase per million reads (FPKM) RNA sequencing data, together with patient-matched clinical data. The cohort is population representative and patients were enrolled between 2010–2015 in South Sweden (PMID:31,434,940) [27].

### Statistical analysis

All experiments were repeated at least 3 times with bars indicating mean ± SD with black and grey circles indicating independent data points in CTRL and CSMD1 groups, respectively. The statistical analyses were performed using GraphPad Prism. The significance level was set at p < 0.05. Details about statistical methods are provided in figure legends. Survival analyses were performed in R version 3.6.1 using the survival package with overall survival (OS) or invasive disease-free survival (IDFS) as clinical endpoints. Survival curves were compared using Kaplan–Meier estimates and the log-rank test. Hazard ratios were calculated through univariable or multivariable Cox regression using the *coxph* R function. In multivariable analyses tumor size (mm), lymph node (LN) status (node-positive / node-negative), and tumor grade were included as covariates. The full available follow-up time was used in calculations.

## Results

### Distinct proteomic signature of CSMD1-expressing MDA-MB-231 breast cancer cells

**Fig. 1:**
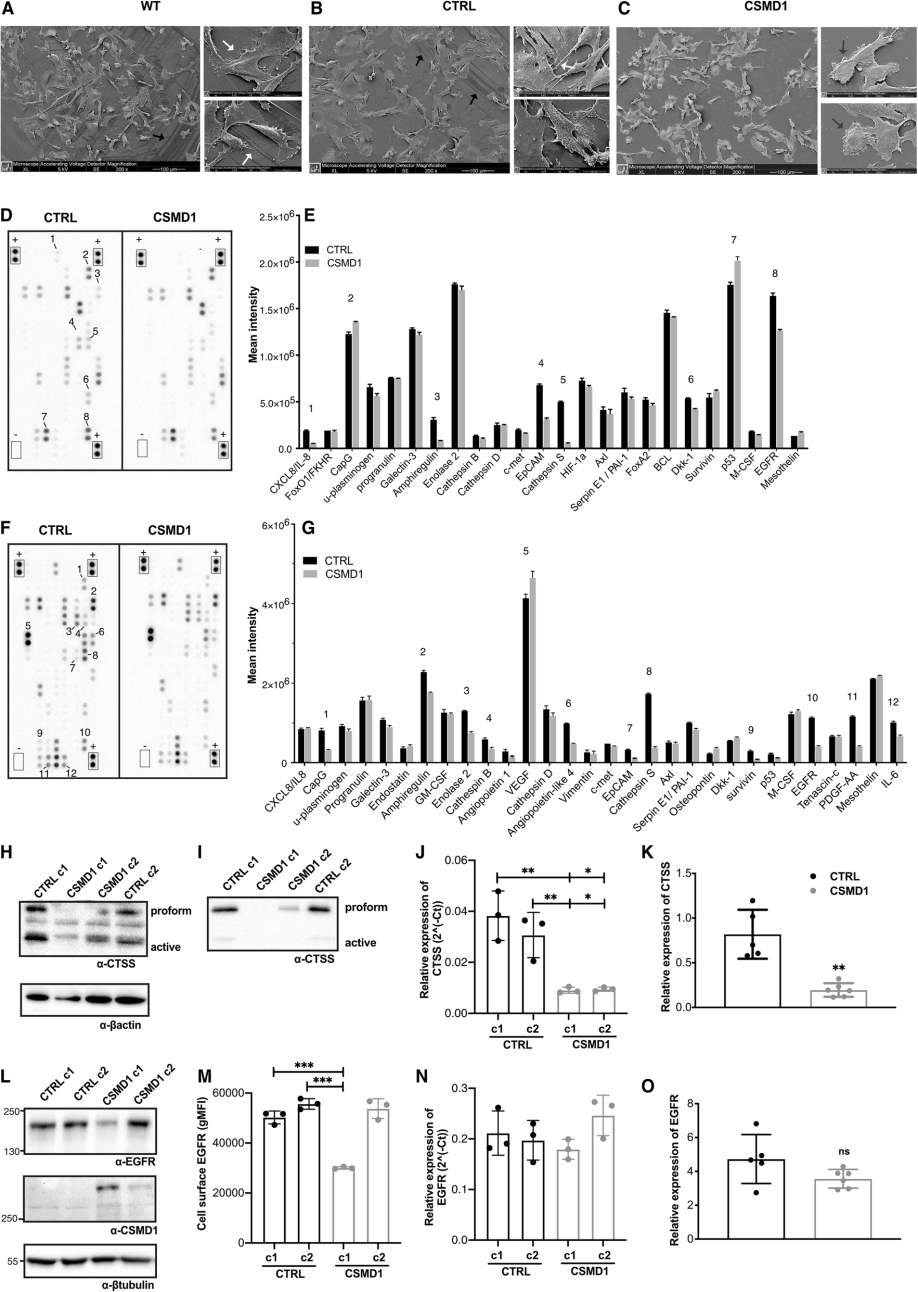
Distinct proteomic signature of CSMD1-expressing MDA-MB-231 BCCs. Scanning electron microscope images of MDA-MB-231 (**A**) WT, (**B**) CTRL and (**C**) CSMD1 BCCs showing distinct morphology of CSMD1-expressing cells. Large panels scale 100 μm. Small panels scale 20 μm. Black arrows indicate the cell “footprints”. White arrows indicate the cytoplasmic protrusions. Dark grey arrows indicate the extracellular material-globular vesicles. Proteome Oncology profiler array—Cancer-related protein analysis of CTRL and CSMD1-expressing MDA-MB-231 cells. **D**, **E** Blots showing the location of proteins and capture antibodies spotted onto the array in duplicates. Positive and negative controls are indicated by + and – adjacent to appropriate spots. Quantification of mean spot pixel intensities of CTRL and CSMD1 cells was plotted in the same order as spotted in the array when analyzing (**D**) cell lysates with the (**E**) corresponding quantifications and (**F**) cell culture supernatants with the (**G**) corresponding quantifications. Numbers correspond to interesting findings in this array. Confirmation of the array: CTSS and EGFR expression in different CTRL and CSMD1 clones of MDA-MB-231 BCCs. Western blot analysis of total cell (**H**) lysates and (**I**) supernatants immunodetection of CTSS with β-actin used as a loading control, and (**J**) mRNA expression levels of *CTSS*. (**L**) Western blot analysis of total cell lysates immunodetecting EGFR and CSMD1 with β-tubulin used as a loading control, (**M**) cell surface EGFR expression assessed by flow cytometry, and (N) mRNA expression levels of *EGFR*. Shown is also mRNA expression of (**K**) *CTSS* and (**O**) *EGFR* in tumors formed in SCID mice injected with MDA-MB-231 CTRL and CSMD1 cells (5 mice in each group). All experiments were repeated at least 3 times. Bars indicate means ± SD. One-way ANOVA Turkey’s multiple comparisons test was used when comparing CSMD1 clones to CTRL clones, and Mann–Whitney comparison test was used when comparing CTRL and CSMD1 groups in tumors formed in vivo (* < 0.05, ** < 0.01, *** < 0.001)

In our previous study, we showed that CSMD1 expression in breast cancer cells (BCCs) decreased their metastatic potential [18]. When analyzed using scanning electron microscopy, MDA-MB-231 wild type (WT) and CTRL cells had a smoother surface, whereas MDA-MB-231 CSMD1 cells produced larger amounts of extracellular material and their surface contained a lot of globular vesicles. In addition, CSMD1-expressing cells were smaller and exhibited diminished formation of filopodia (cytoplasmic protrusions). Further, we only observed cell footprints indicating movement in WT and CTRL cells in contrast to the CSMD1 “amotile” cells (Figs. 1A-C). We hypothesized that this distinct morphological architecture of CSMD1-expressing cells is a result of altered cellular protein expression and remodeling, characteristic for oncogenic transformation and progression of cancer. Therefore, total protein cell extracts from MDA-MB-231 CTRL and CSMD1-expressing cells (Figs. 1D-E), as well as their corresponding secretomes (supernatants) (Figs. 1F-G), were analyzed using Human Proteome Oncology Arrays. Overall, the results indicated several possible mediators of CSMD1 action, involving growth factor receptors and their ligands (EGFR/AREG, PDGF-AA, VEGF), cysteine proteases (CTSS and CTSB), as well as adhesion and actin remodeling molecules (EpCAM, CapG) (Figs. 1E-G). Cathepsin S (CTSS) showed the greatest decrease in expression in MDA-MB-231 CSMD1 cells, followed by Epidermal Growth Factor Receptor (EGFR) and its ligand amphiregulin (AREG), as compared with CTRL cells. Consistent with our observations of altered morphology of the MDA-MB-231 CSMD1 cells, adhesion and actin remodeling molecules in the array (EpCAM, CapG) were downregulated in CSMD1-expressing cells. Other proteins, which also showed altered expression included proinflammatory cytokines, interleukins IL-8 and IL-6. On the other hand, the expression of p53 tumor suppressor was upregulated in MDA-MB-231 CSMD1 cells, compared to CTRL.

The protein array data were confirmed using different experimental approaches. First, CTSS expression was assessed in lysates (Fig. 1H) and corresponding supernatants (Fig. 1I) of several clones of CSMD1-expressing MDA-MB-231 cells, using Western blotting. CTSS expression and secretion from CSMD1-expressing cells was downregulated. Accordingly, CTSS mRNA levels were also significantly suppressed in MDA-MB-231 CSMD1 in comparison with CTRL cells (Fig. 1J), and this observation was confirmed in tumors formed in SCID mice injected with MDA-MB-231 CTRL and CSMD1 cells (Fig. 1K).

In addition, EGFR protein expression was assessed in lysates of several clones of CSDM1-expressing MDA-MB-231 cells using Western blotting. We found that EGFR expression was negatively correlated with CSMD1 expression in the different clones. The clone c1, highly expressing CSMD1, exhibited the lowest expression of EGFR, while the other CSMD1 clone expressing lower levels of CSMD1 exhibited comparable EGFR expression with the CTRL clones (Fig. 1L). We further confirmed this observation detecting cell-surface EGFR using flow cytometry (Fig. 1M), while the levels of mRNA coding for *EGFR* (Fig. 1N), and its ligands such as *EGF*, *TGFα* and *AREG* were not altered in the CSMD1 clones in comparison with the CTRL cells (Supplementary Fig. 1A-C). Of note, as we have seen previously, the mRNA levels of *CSMD1* seem to correlate with its protein expression in the different clones [28]. Accordingly, there was no difference in *EGFR* mRNA expression in tumors formed in SCID mice injected with MDA-MB-231 CTRL and CSMD1 cells (Fig. 1O). Further, mRNA expression databases for breast cancer patients available online, also did not indicate correlation between mRNA levels of EGFR and CSMD1 (Supplementary Fig. 1D & E). Therefore, the observed difference on the protein level may depend on post-translational mechanisms such as receptor trafficking or degradation.

### CSMD1 interacts directly with EGFR

**Fig. 2:**
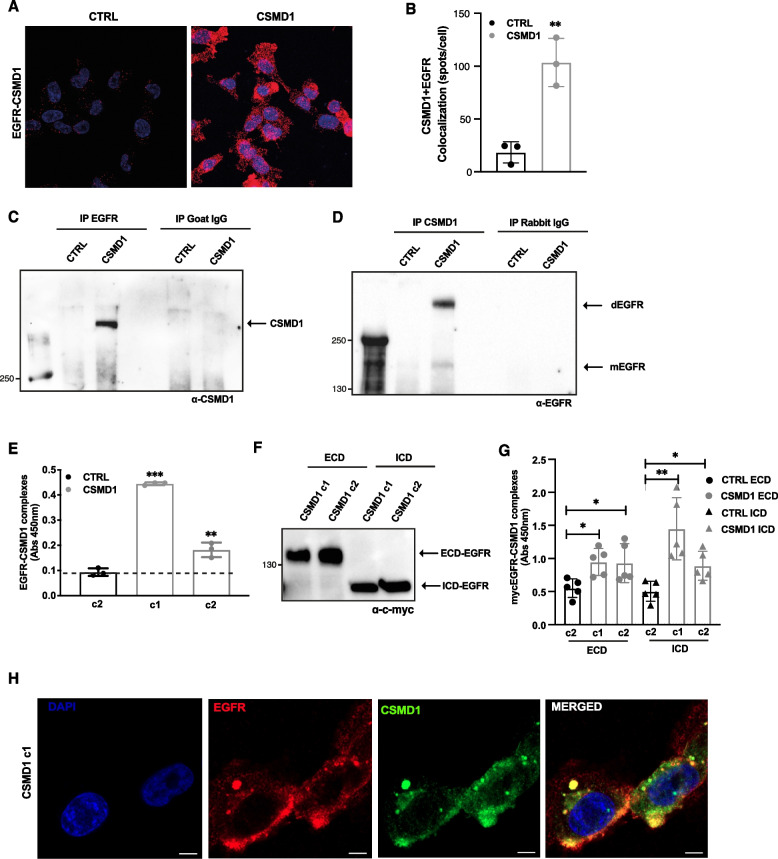
CSMD1 interacts with EGFR. **A** Representative confocal z-stacked maximum projection images of PLA using MDA-MB-231 CTRL and CSMD1 cells. **B** Quantification of the dots per cell showing number of interactions between CSMD1 and EGFR. Binding between CSMD1 and EGFR was confirmed using co-immunoprecipitation (**C**) Cell lysates were immunoprecipitated using antibodies against EGFR and corresponding IgG control followed by immunodetection of CSMD1 protein. **D** Cell lysates were immunoprecipitated using antibodies against CSMD1 and corresponding IgG control followed by immunodetection of EGFR protein. **E** In ELISA setup an anti-EGFR antibody was used to capture the EGFR protein complexes lysed under non-denatured conditions and an anti-CSMD1 antibody applied to detect the EGFR-CSMD1 interactions. **F** MDA-MB-231 cell expressing CSMD1 and the control cells were transiently transfected with plasmid constructs that express extracellular domain of EGFR (ECD) and intracellular domain of EGFR (ICD) tagged with c-Myc. Representative blot detecting c-myc and confirming efficient transfection of the cells is shown. **G** The anti-c-myc capturing antibody was immobilized in ELISA plates, incubated with cell lysates followed by an anti-CSMD1 antibody to detect the myc-ECD-EGFR-CSMD1 and myc-ICD-EGFR-CSMD1 complexes. **H** Colocalization of CSMD1 and EGFR. MDA-MB-231 CSMD1-expressing cells (c1 clone) were fixed and stained with anti-EGFR (red) and anti-CSMD1 (green). DAPI (blue) was used to stain the nuclei. Scale bars 10 μm. All experiments were repeated at least 3 times with bars indicating mean ± SD, black and grey circles correspond to independent data points for CTRL and CSMD1 groups, respectively. Unpaired t-test was used when comparing 2 samples, one-way ANOVA Dunnett’s multiple comparisons test was used when compared 3 or more samples, and two-way ANOVA Bonferroni’s multiple comparisons test was used when comparing 3 or more groups with 2 variables (* < 0.05, ** < 0.01, *** < 0.001, **** < 0.0001)

EGFR (also known as ERBB or HER1) is a member of the HER cell-surface receptor tyrosine kinase family. Considering the localization of CSMD1 on the cell surface, we hypothesized that CSMD1 may act as a regulator of EGFR function. Indeed, a proximity ligation assay (PLA) indicated that CSMD1 interacted with EGFR in the MDA-MB-231 CSMD1 cells (Fig. 2A&B). Furthermore, lysates of CTRL and CSMD1 MDA-MB-231 cells were prepared in minimally denaturing conditions and analyzed by co-immunoprecipitation. CSMD1 was specifically detected in EGFR-precipitates by Western blotting, whereas no signal was observed either in the negative control (CTRL MDA-MB-231), or when isotype control antibodies were used (Fig. 2C). Furthermore, in immunoprecipitated CSMD1-complexes we detected a weaker signal approximately corresponding to the size of EGFR monomer (175 kDa) and a stronger signal of EGFR dimers, while no such signals were detected with the corresponding isotype control antibodies (Fig. 2D). The direct binding between CSMD1 and EGFR was further confirmed using ELISA in which complexes were captured with immobilized anti-EGFR antibodies and detected with anti-CSMD1 antibodies (Fig. 2E). The EGFR-CSMD1 interaction was detected by ELISA in two different clones with varying overexpression levels of CSMD1. Furthermore, CTRL and CSMD1-expressing cells were transiently transfected with the Myc-tagged extracellular domain of EGFR (ECD) and Myc-tagged intracellular domain of EGFR (ICD). The efficient transfection of ECD-EGFR and ICD-EGFR constructs and subsequent expression was confirmed by western blot using an anti-c-myc antibody (Fig. 2F). The interaction of CSMD1 with both ECD-EGFR, as well as ICD-EGFR in two clones differing in CSMD1 expression levels was detected by ELISA, in which complexes were captured with immobilized anti-myc antibodies and detected with anti-CSMD1 antibodies (Fig. 2G). Finally, using confocal microscopy, we documented that CSMD1 co-localizes with EGFR on the cell surface as well as in spotted structures resembling trafficking vehicles (Fig. 2H).

### EGFR signaling is decreased in the presence of CSMD1

To determine EGFR activation in the presence of CSMD1, EGFR was immunoprecipitated in EGF-stimulated CTRL and CSMD1 expressing MDA-MB-231 cells and overall phosphorylation levels were examined by immunoblotting for phosphotyrosine and phosphoserine residues. As expected, EGF induced overall tyrosine and serine phosphorylation of EGFR in the CTRL clones. In contrast, CSMD1-expressing clones exhibited markedly less overall tyrosine and serine phosphorylation in both CSMD1-expressing clones (Supplementary Fig. 1F-I).

**Fig. 3:**
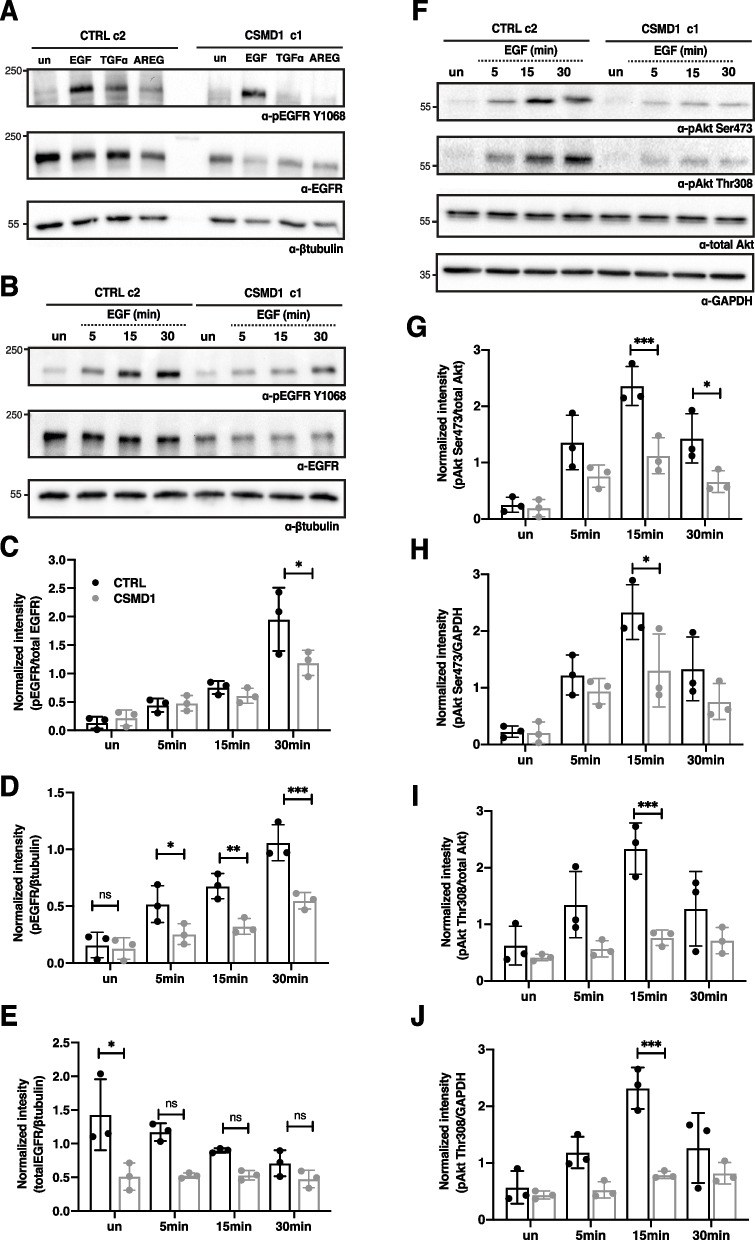
EGFR signaling cascade in the presence of CSMD1. **A** Representative western blots of total lysates CTRL and CSMD1 MDA-MB-231 cells immunodetecting phosphorylated EGFR at the residue Y1068 (pEGFR Y1068) and total EGFR with β-tubulin used as a loading control upon stimulation with EGF (25 ng/mL), TGF-α (25 ng/mL) and AREG (100 ng/mL) for 30 min. CTRL and CSMD1 MDA-MB-231 cells were serum starved for 2 h and then treated with 25 ng/mL EGF for the indicated time points (5, 15 and 30 min). Unstimulated cells were also included in the experiment. **B** Immunoblot analysis of phosphorylated EGFR at the residue Y1068, total EGFR and β-tubulin used as a loading control. **C** Densitometry of pEGFR Y1068 normalized to total EGFR. **D** Densitometry of pEGFR Y1068 normalized to β-tubulin, and (**E**) densitometry of total EGFR normalized to β-tubulin. **F** Immunoblot analysis of phosphorylated Akt at the residues Ser473 (pAkt Ser473) as well as Thr308 (pAkt Thr308), total Akt and GAPDH used as a loading control. **G** Densitometry of pAkt Ser473 normalized to total Akt. **H** Densitometry of pAkt Ser473 normalized to GAPDH, (**I**) densitometry of pAkt Thr308 normalized to total Akt and (**J**) densitometry of pAkt Thr308 normalized to GAPDH. All experiments were repeated at least 3 times with bars indicating mean ± SD, black and grey circles correspond to independent data points for CTRL and CSMD1 groups, respectively. A two-way ANOVA Bonferroni’s multiple comparisons test was used when comparing 3 or more groups with 2 variables (* < 0.05, ** < 0.01, *** < 0.001, **** < 0.0001)

Apart from EGF, EGFR can be activated by numerous structurally related ligands with different binding affinities and subsequent signaling kinetics [29, 30]. Thus, we evaluated the effect of CSMD1 expression on EGFR phosphorylation at residue Y1068, known to correlate with EGFR kinase activation, while stimulating with two high affinity ligands EGF (25 ng/mL) and TGFα (25 ng/mL), as well as a weaker affinity ligand, AREG (100 ng/mL). In all cases, CSMD1 expression in the MDA-MB-231 cells decreased the activation of EGFR at Y1068 in response to its natural ligands, especially TGFα and AREG (Fig. 3A). EGF-induced EGFR activation increased in a time-dependent manner in the CTRL cells, in contrast with CSMD1-expressing cells in which EGFR activation was delayed and did not reach the same levels as in CTRL cells, after 30 min stimulation (Fig. 3B-D). In the case of CTRL cells, EGFR expression was downregulated during EGF stimulation, whereas in CSMD1-expressing cells the EGFR level was stable and downregulated in a ligand-independent manner (Fig. 3B & E).

Active EGFR initiates several signaling cascades. Thus, we next assayed the activation status of key downstream effectors arising upon EGF stimulation. Specifically, in CSMD1-expressing cells we detected a diminished AKT phosphorylation at both Ser473 and Thr308 residues at 15 min and 30 min EGF stimulation, in comparison with CTRL cells (Fig. 3F-J). This indicates a possible effect of CSMD1 in PI3K through the inhibition of EGFR activation.

Taken together, the EGF-induced EGFR signaling axis is affected by the presence of CSMD1, while it appears to be possibly accompanied by an altered endocytic trafficking fate of the receptor.

### CSMD1 governs EGFR endosomal trafficking fate

Binding of EGF to EGFR leads to internalization of the receptor and trafficking via the endocytic pathway. Binding assays with ^125^I-labeled EGF indicated that CSMD1-expressing cells exhibit a similar binding affinity to EGF as CTRL cells (Supplementary Fig. 1J).

**Fig. 4:**
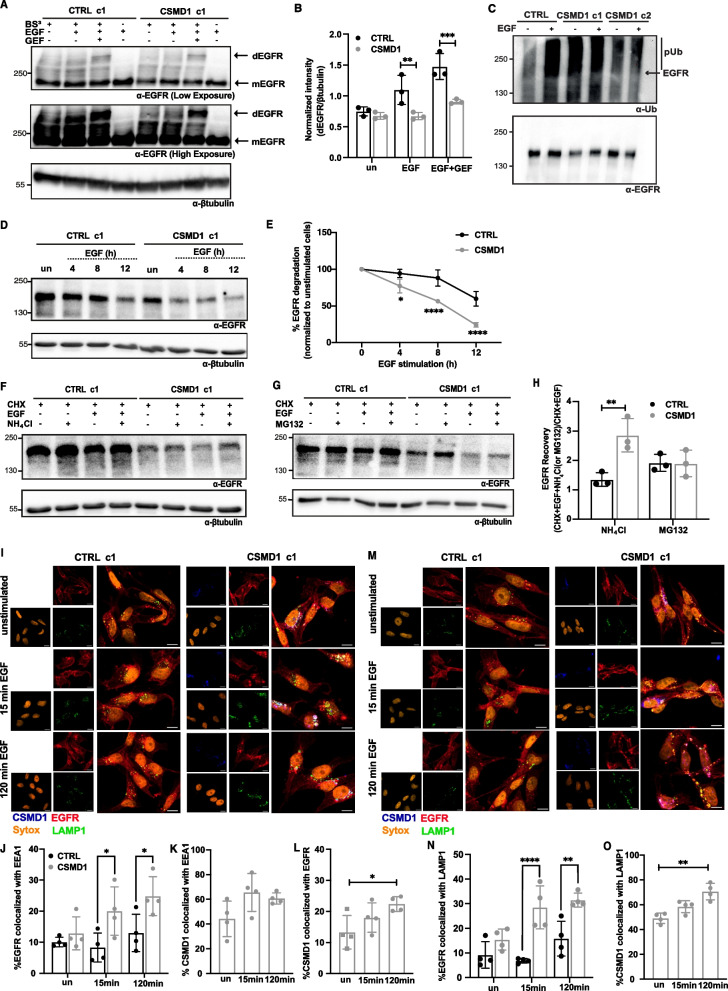
EGFR endocytic trafficking in the presence of CSMD1. **A** Detection of EGFR dimers upon EGF (25 ng/ml for 15 min) and GEF (10 μM) stimulation followed by crosslinking with BS^3^. **B** Densitometry of dimers of EGFR normalized to βtubulin (**C**) CSMD1 triggers EGFR ubiquitination which was detected using EGFR immunoprecipitation followed by immunoblotting with anti-Ub antibody. Representative blots from three independent experiments are shown. **D** Representative immunoblots of EGFR levels in lysates of MDA-MB-231 CTRL and CSMD1 overexpressing clonal cells, pre-treated with translation inhibitor cycloheximide (CHX) (100 μg/mL) for 2 h and treated with EGF (25 ng/ml) for 0, 4, 8, 12 h (h). **E** Quantification of EGFR levels in MDA-MB-231 cells plotted against time. Representative immunoblots of EGFR levels in CTRL and CSMD1 overexpressing cells, pre-treated with CHX (100 μg/mL) for 2 h followed by treatment with 25 ng/mL EGF in the presence of (**F**) NH_4_Cl and (**G**) MG132 for 12 h. **H** Quantification of EGFR recovery in both NH_4_Cl or MG132 treated CSMD1 and CTRL cells. **I** Representative confocal images of CTRL and CSMD1 BCCs co-stained for EGFR (red), CSMD1 (blue), EEA1 (green), nucleus (SYTOX orange). Quantification of colocalization of (**J**) EGFR-EEA1, (**K**) EEA1-CSMD1 and (**L**) CSMD1-EGFR complexes. **M** Representative confocal images of CTRL and CSMD1 BCCs co-stained for EGFR (red), CSMD1 (blue), LAMP1 (green), nucleus (SYTOX orange). Quantification of colocalization of (**N**) EGFR-LAMP1 and (**O**) LAMP1-CSMD1 complexes. Scale bars 5 μm. All experiments were repeated at least 3 times with bars indicating mean ± SD, grey circles correspond to independent data points for CTRL and CSMD1 groups, respectively. A two-way ANOVA Bonferroni’s multiple comparisons test was used when comparing CTRL and CSMD1 groups (* < 0.05, **** < 0.0001). A one-way ANOVA Dunnetts’s multiple comparisons test was used when comparing CSMD1 cells in different time-points (* < 0.05, **** < 0.0001)

Since we observed lower EGFR levels and altered dynamics of EGFR downstream signaling in CSMD1-expressing cells, we investigated whether CSMD1 contributes to the receptor turnover and stability. First, we monitored EGFR dimerization upon EGF stimulation in CTRL and CSMD1 MDA-MB-231 cells by cross-linking the cell surface proteins and subsequently detecting the formed EGFR dimers induced by EGF stimulation. EGFR monomers and dimers were detected by western blotting with antibodies detecting EGFR, while treatment with Gefitinib (GEF), a tyrosine kinase inhibitor, was used as a positive control, as it is reported in the literature to induce EGFR dimerization [26]. EGF stimulation induced EGFR dimerization in CTRL cells, whereas CSMD1-expressing cells exhibited a decreased rate of EGFR dimerization, even upon treatment with GEF (Fig. 4A & B).

EGFR signaling can also be modulated by the post-translational modification ubiquitination. To address EGFR trafficking in the presence of CSMD1, whole lysates of MDA-MB-231 CTRL and CSMD1 cells in denaturing and minimally denaturing conditions were subjected to immunoprecipitation of EGFR, and ubiquitination was monitored using immunoblotting for ubiquitin (Ub). High levels of EGFR ubiquitination were detected in the presence of CSMD1, both in unstimulated and stimulated conditions (Supplementary Fig. 2A). In addition, when analyzing minimally denaturing lysates, we observed a high recruitment of Ub-tagged proteins to EGFR in the presence of CSMD1 (Fig. 4C).

The two fates of EGFR after the binding of EGF are either degradation or recycling [31]. Therefore, we firstly investigated if CSMD1 affects the EGF-induced EGFR degradation by treating MDA-MB-231 CTRL and CSMD1 overexpressing cells with EGF for 0, 4, 8, 12 h followed by measurements of total EGFR levels in the obtained lysates. To eliminate the impact of de-novo EGFR synthesis, both CTRL and CSMD1 cells were pre-treated with the translational inhibitor cycloheximide (CHX) before and during the EGF stimulation. CSMD1 increased the EGFR degradation rate, as in CSMD1-expressing cells the EGFR half-life (8 h) was shorter than in CTRL cells (12 h; Fig. 4D &E). On the other hand, the internalization rate of ^125^I-labeled EGF was comparable between CTRL and CSMD1-expressing cells (Supplementary Fig. 2B). Therefore, we hypothesized that CSMD1 may direct EGFR towards degradation by ubiquitination, thus modulating its trafficking.

Proteasomal and lysosomal degradation systems are the main drivers of EGFR degradation. To evaluate which system is responsible for the increased EGFR degradation in CSMD1-expressing cells, we performed similar degradation experiments as described above in the presence of a lysosomal inhibitor (NH_4_Cl), or proteasomal inhibitor (MG132). Our results revealed that increased EGFR degradation in the presence of CSMD1 was reverted only when lysosomal activity was inhibited (Fig. 4F), while proteasome inhibition did not alter EGFR degradation levels (Fig. 4G). EGFR recovery in the presence of inhibitors was also calculated, highlighting the higher EGFR recovery upon treatment with NH_4_Cl in the CSMD1-expressing cells compared to CTRL, whereas no effect of MG132 on EGFR recovery was observed (Fig. 4H). To further support this observation, we investigated EGFR localization by confocal microscopy in CTRL and CSMD1 MDA-MB-231 cells, using early and late endosomal markers, Early Endosome Antigen 1 (EEA1) (Fig. 4I) and Lysosomal-associated membrane protein 1 (LAMP1) (Fig. 4M), respectively. Following 15 min and 120 min EGF stimulation, colocalization between EGFR and/or EEA1 and LAMP1 was barely observed in CTRL cells, while it was increased in CSMD1-expressing cells, especially in the case of late endosomal marker, suggesting that CSMD1 plays a fundamental role in EGFR trafficking (Fig. 4J & N). In addition, cell fractionation into cytosol and membrane extracts revealed a shift of EGFR from the membrane to the cytosolic fractions in CSMD1-expressing cells compared to CTRL cells, in both unstimulated and EGF-stimulated conditions (Supplementary Fig. 2C & D), supporting increased internalization/degradation in CSMD1-expressing cells. On the other hand, CSMD1 itself highly colocalized with both EEA1 and LAMP1, but only in the case of LAMP1 was the colocalization significantly increased upon 120 min EGF stimulation (Fig. 4K & O). Interestingly, EGFR-CSMD1 colocalization was also significantly increased after 120 min EGF stimulation (Fig. 4L). In addition, a stronger triple colocalization of EGFR-CSMD1-EEA1 and weaker EGFR-CSMD1-LAMP1 colozalisation were detected in CSMD1-expressing cells.

Key findings reported in this study were confirmed in two additional triple negative breast cancer cell lines, BT-20 and MDA-MB-468. Firstly, we documented CSMD1-EGFR interaction in BT-20 cells by co-immunoprecipitation (Supplementary Fig. 3A). However, in contrast with MDA-MB-231 cells, in immunoprecipitated CSMD1-complexes we detected a stronger signal approximately corresponding to the size of EGFR monomer and a weaker signal of EGFR dimers, while no such signals were detected with the corresponding isotype control antibodies. In BT-20 and MDA-MB-468 cells, even though EGF-induced EGFR phosphorylation was not markedly affected (Supplementary Fig. 3B-E & Supplementary Fig. 4A-D), the PI3K-AKT cascade was also inhibited by CSMD1 upon EGF stimulation, similar to MDA-MB-231 BBCs. Specifically, activation of AKT at the Ser473 residue was less pronounced in CSMD1-expressing BT-20 and MDA-MB-468 cells than CTRL cells, at shorter time points (Supplementary Fig. 3F-H & Supplementary Fig. 4E-G). In addition, EGF-induced EGFR degradation in BT-20 and MDA-MB-468 CSMD1 cells was faster in comparison to CTRL cells, suggesting that CSMD1 acts on EGFR action via multiple mechanisms (Supplementary Fig. 3I & J & Supplementary Fig. 4H-I).

### CSMD1 expression increases sensitivity to chemotherapy

**Fig. 5:**
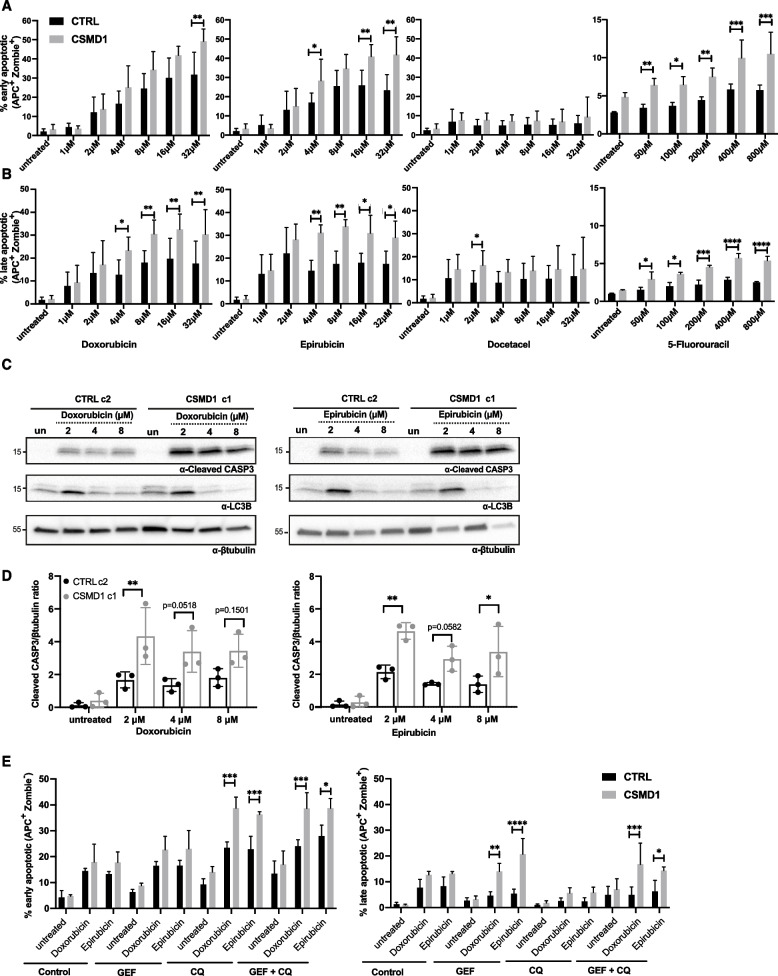
CSMD1-expressing BCCs are sensitive to chemotherapy. MDA-MB-231 CTRL and CSMD1 cells were treated with different chemotherapy agents for 48 h, and apoptosis was monitored using annexin V staining while live/dead cell discrimination was performed with Zombie Aqua staining, both using flow cytometry. Bar graphs showing percentages of (**A**) early and (**B**) late apoptotic cells. **C** Immunoblot analysis of cleaved caspase-3 and LC3B upon treatment with different chemotherapy agents; β-tubulin was used as a loading control. **D** Densitometry analysis of cleaved caspase-3 normalized to β-tubulin. **E** MDA-MB-231 CTRL and CSMD1 cells were pre-treated with GEF, QC alone and combination of both for 24 h, following doxorubicin and epirubicin (1 μM) treatment for 48 h. Bar graphs showing percentages of early and late apoptotic cells. All experiments were repeated at least 3 times with bars indicating mean ± SD, grey circles correspond to independent data points for CTRL and CSMD1 groups, respectively. A two-way ANOVA Bonferroni’s multiple comparisons test was used when comparing CTRL and CSMD1 groups in different concentration of chemotherapy drugs or treatments

We hypothesized that CSMD1-bearing tumors are sensitive to common breast cancer chemotherapy agents and that CSMD1 expression could therefore be used as a predictive marker of response to therapy. To address this we treated MDA-MB-231 CTRL and CSMD1 cells with increasing doses of chemotherapy agents and monitored drug-induced apoptosis by annexin V staining, and live/dead cell discrimination with Zombie aqua staining, using flow cytometry (Fig. 5A & B). The chemotherapy agents tested, doxorubicin (a topoisomerase activity inhibitor), epirubicin (an anthracycline drug), docetaxel (a microtubule inhibitor), as well as 5-fluorouracil (pyrimidine analog antimetabolite) were not effective in killing the TNBC MDA-MB-231 CTRL cells in the range of concentrations used, as documented by the percentage of late apoptotic cells. In contrast, CSMD1 MDA-MB-231 expressing cells were sensitive to doxorubicin-, epirubicin- and 5-fluorouracil-induced apoptosis in a dose dependent manner, while docetaxel was less effective, showing a trend in the lower doses and a significant killing effect in the highest dose used (Fig. 5B). Additionally, caspase-3 activity, monitored by the levels of cleaved caspase-3, was significantly upregulated in the CSMD1 cells treated with doxorubicin and epirubicin in comparison with CTRL cells, corresponding well with the increased observed numbers of apoptotic cells (Fig. 5C & D). In line with this observation, BT-20 and MDA-MB-468 CSMD1-overexpressing cells also exhibited increased sensitivity to the chemotherapy drugs doxorubicin and epirubicin (Supplementary Fig. 3 K & Supplementary Fig. 4J).

In breast cancer, several mechanisms of drug resistance are known, including autophagy and drug efflux/uptake. In our experimental setup, no statistical difference was observed between CTRL and CSMD1 BCCs in autophagy induction upon chemotherapy treatment, as monitored by conversion of LC3BI to LC3BII (Fig. 5C). However, a doxorubicin efflux assay indicated that drug efflux activity or uptake were impaired in the presence of CSMD1, resulting in increased intracellular drug availability (Supplementary Fig. 5A&B). Even though EGFR overexpression is a common feature of TNBC, tyrosine kinase inhibition monotherapy is not effective. Pretreatment of the BCCs with the tyrosine kinase inhibitor GEF followed by low doses of doxorubicin or epirubicin (1 μM) did not overcome resistance of CTRL cells to chemotherapy, while CSMD1-expressing cells exhibited a significant increase in the percentage of late apoptotic cells, comparable to the highest concentration of chemotherapy agent used in the monotherapy experimental setup (Fig. 5E). This effect was not reversed when blocking autophagy with chloroquine, and pretreatment with GEF did not affect autophagy levels (Supplementary Fig. 5C). Taken together, CSMD1 inhibits chemoresistance of BBCs by affecting intracellular drug availability, rather than autophagy.

**Fig. 6:**
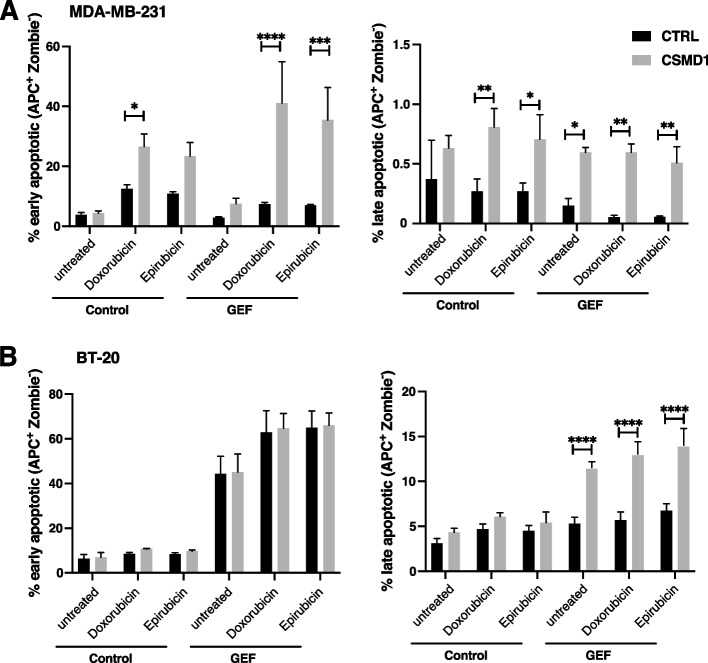
CSMD1-expressing tumorspheres are more sensitive to chemotherapy agents. **A** MDA-MB-231 and (**B**) BT-20 tumorspheres were pre-treated with 35 μM of GEF followed by 2 μM of doxorubicin or epirubicin for two days (MDA-MB-231) or 8 μM of doxorubicin and epirubicin for one day (BT-20). Early and late apoptotic cells were detected using Annexin V and Zombie Aqua staining. Bar graphs showing percentages of early and late apoptotic cells. All experiments were repeated at least 3 times with bars indicating mean ± SD, grey circles correspond to independent data points for CTRL and CSMD1 groups, respectively. A two-way ANOVA Bonferroni’s multiple comparisons test was used when comparing CTRL and CSMD1 groups in different treatments (* < 0.05, ** < 0.01, *** < 0.001)

To strengthen our findings and provide a better representation of the in vivo environment [32], we generated uniform tumorspheres of MDA-MB-231 and BT-20 cells. Similar to the two dimensional cell culture, CSMD1-expressing cells were more sensitive to chemotherapy treatment, especially in the case of MDA-MB-231 (Fig. 6Α), whereas BT20 cells were more resistant in general (Fig. 6B). Further, pretreatment of the CSMD1-expressing tumorspheres with the tyrosine kinase inhibitor GEF followed by low doses of doxorubicin or epirubicin overcame the resistance of CTRL cells to chemotherapy, especially in the case of BT-20 cells where only the GEF pretreated CSMD1-expressing cells exhibited a significant increase in the percentage of late apoptotic cells (Fig. 6B). Similar observations were documented for MDA-ΜΒ-231 CSMD1 expressing cells, presenting a markedly higher percentage of early apoptotic cells in the GEF pretreated cells in contrast with CTRL cells (Fig. 6A). In conclusion, CSMD1 protein expression rendered tumorspheres pretreated with EGFR inhibitor more sensitive to chemotherapy.

### *CSMD1* gene expression levels and association with outcome in breast cancer patients

**Fig. 7:**
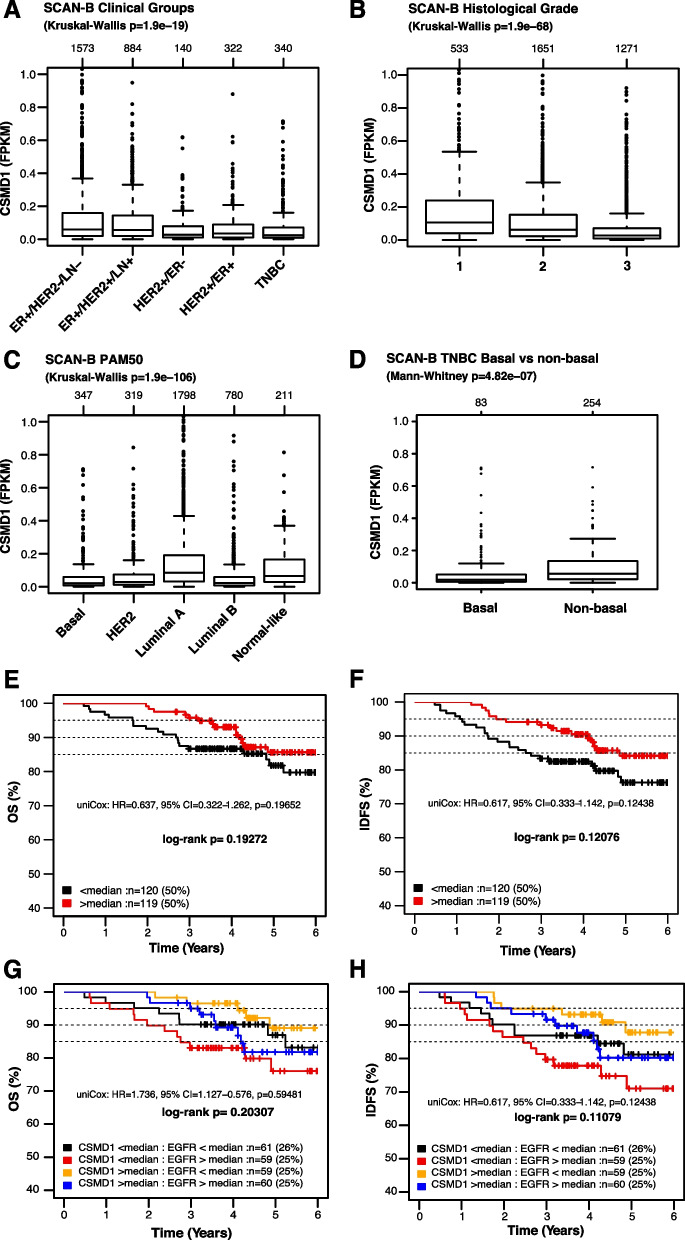
Gene-set tumor analysis of *CSMD1* expression using 3520 breast cancer patients SCAN-B cohort. Box plot of *CSMD1* gene expression for tumor samples of SCAN-B cohort stratified according to (**A**) clinical groups, (**B**) tumor grade (Nottingham Histological Grade) (**C**) PAM50 subtypes and (**D**) box plot of *CSMD1* gene expression for tumor samples of SCAN-B cohort subset of TNBC stratified according to basal and non-basal like properties. Kaplan–Meier analysis, using (**E**) OS and (**F**) IDFS as endpoints, for TNBC patients treated with chemotherapy (n = 239) stratified into the two quantiles based on *CSMD1* median gene expression level. Kaplan–Meier analysis, using (**G**) OS and (**H**) IDFS as endpoints, for TNBC patients treated with chemotherapy (n = 239) stratified into the four quantiles based on *CSMD1* and *EGFR* median gene expression level

In our previous study, we showed that *CSMD1* mammary gene expression is dramatically decreased in breast cancer patients compared to normal mammary tissue [28]. In the current study, we expanded our mRNA analyses to a modern population-based cohort, the Swedish Cancerome Analysis Network – Breast (SCAN-B), consisting of 3520 RNA sequenced primary breast cancer patients as reported by [27]. In accordance with previous observations, *CSMD1* gene expression (FPKM) was generally low across SCAN-B patients with no bimodal patterns. Of note, this low, unimodal expression makes cut-offs for gene expression defined groups arbitrary (Supplementary Fig. 6A & B). Moreover, the *CSMD1* gene expression profile across clinical subgroups (ER, HER2, and LN status, as well as histological grade) and molecular PAM50 subtypes (Basal, HER2, Luminal A, Luminal B and normal-like) of SCAN-B patients was investigated. In all cases, low transcriptional variation was observed across the different subgroups, while there was a range of outliers within each subgroup. Still, *CSMD1* gene expression levels showed significant differences between certain clinical subgroups, with e.g. lower levels in *HER2*-amplified disease and TNBC compared to luminal disease, lower levels in grade 3 tumors compared to grade 1 and 2 tumors, and lower levels in the basal-like, HER2-enriched, and luminal B PAM50 subtypes compared to luminal A tumors (Kruskal–Wallis test p < 0.00001, Fig. 7A-B). *CSMD1* expression differences were also observed within clinical subgroups, e.g., in TNBC lower mRNA expression was observed in PAM50 basal-like TNBC cases compared to non-basal-like TNBC (Mann–Whitney test p < 0.00001, Fig. 7C). Difference in *CSMD1* expression between basal and non-basal in TNBC was mirrored by a similar trend for EGFR (Mann–Whitney test p = 0.008, Supplementary Fig. 6C).

To test the association of *CSMD1* gene expression with patient outcome after adjuvant standard-of-care chemotherapy in TNBC, we stratified the 239 adjuvant-treated TNBC patients according to the median expression of *CSMD1* and analyzed the two groups using overall survival (OS) and invasive disease-free survival (IDFS) as clinical endpoints. For both endpoints, higher gene expression of *CSMD1* tended to be associated with better prognosis, although both log-rank p-values and univariable Cox regression analysis did not reach statistical significance (Fig. 7E & F).

To assess the independent prognostic value of *CSMD1* gene expression in adjuvant treated TNBC patients, we performed multivariable Cox regression analysis adjusting for tumor size (≤ 20 mm, > 20 mm), tumor grade (2, 3), and lymph node status (N0, N +) using OS and IDFS as the clinical endpoints. *CSMD1* gene expression above the median did not reach statistical significance (p < 0.05), although similar trends, as in the Kaplan–Meier and univariable Cox analyses, of improved OS (HR = 0.63, 95% confidence interval (CI): 0.32–1.3, p = 0.201) and IDFS (HR = 0.63, 95% confidence interval (CI): 0.33–1.1, p = 0.123) were observed.

To test the association of *CSMD1* and *EGFR* gene expression with outcome after adjuvant standard-of-care chemotherapy in TNBC, we further stratified the 239 adjuvant-treated TNBC patients according to the median expression of *CSMD1* and *EGFR*, and analyzed the four groups using overall survival (OS) and invasive disease-free survival (IDFS) as clinical endpoints. For both endpoints, higher gene expression of *CSMD1* together with low gene expression of *EGFR* tended to be associated with better prognosis, although both log-rank p-values and univariable Cox regression analysis did not reach statistical significance. In contrast, lower gene expression of *CSMD1* together with high gene expression of *EGFR* tended to be associated with worse prognosis (Fig. 7G & H).

Collectively, in accordance with our *in vitro* data, a trend of higher chemosensitivity in CSMD1-expressing tumors appears to be evident in a population-based cohort of TNBC. Moreover, *CSMD1* gene expression differed between basal-like and non-basal-like TNBC, suggesting a connection to underlying biological differences between these subgroups. Noteworthy, *EGFR* expression together with *CSMD1* further characterizes subsets of BC patients with varying chemosensitivity.

## Discussion

TNBC is an aggressive breast cancer subtype, characterized by the lack of expression of estrogen receptor (ER), progesterone receptor, and human epidermal growth factor receptor 2 (HER2). There are no clinically approved targeted therapies for TNBC, and thus, there is an urgent need to identify potent, highly effective therapeutic targets. EGFR is of immediate medical and biological importance due to its well-established roles in developmental biology, tissue homeostasis, and cancer [33]. Overexpression of EGFR has been reported in 15–20% of all breast carcinomas and in 50–70% of TNBCs [34, 35]. It is known that breast cancers with high EGFR expression are more aggressive, larger in size and more capable to metastasize to the lymph nodes and brain. Additionally, patients with EGFR-positive tumors have a worse overall, disease free and post-relapse survival after hormonal and/or chemotherapy, lacking prognostic indicators [36, 37]. However, EGFR-targeted therapy has poor performance in TNBC [37]. In the current study, we report for the first time the direct interaction of EGFR with the tumor suppressor CSMD1, which leads to attenuation of EGFR signaling due to altered trafficking of the receptor. Importantly, CSMD1-expressing cells are as a result more sensitive to chemotherapy.

To date, we and others have presented evidence that CSMD1 acts as a tumor suppressor in breast cancer, but molecular mechanisms underlying this function of the protein have not been elucidated [28, 38]. Since CSMD1 is a poorly studied protein, we resorted to general screens such as proteome oncology arrays to obtain insights into its cellular effects. Scanning electron microscopy revealed large phenotypic changes in CSMD1-expressing cells that translated into a distinct proteomic signature, rendering them less aggressive. Specifically, proteome profiling showed significant changes in expression of CapG and EpCAM that are both involved in organization of the actin cytoskeleton, which could be related to an observed effect on cell motility [39, 40]. In addition, CTSS protein expression was decreased by CSMD1, reflecting the low metastatic potential of these cells. Recently, CTSS was introduced as a potential biomarker in TNBC, while mechanistically contributing to BCC's ability to metastasize to the brain via a Src dependent mechanism [41, 42]. In this study, we focused on the relation between CSMD1 and EGFR, considering the importance of EGFR in TNBC. The motivation behind this was that in the presence of CSMD1, we documented that EGFR overall expression, as well as cell surface expression, was markedly altered, even though its gene expression/mRNA level was not affected.

Upon binding of the ligand, EGFR, which is otherwise in a monomeric, inactive state, aggregates at the cell surface forming homodimeric or heterodimeric complexes, usually with other members of HER family or other receptors. Collectively in this study, several methods were used to show direct interaction between CSMD1 and EGFR, which appears to be mediated by both the extracellular and intracellular portions of EGFR. That interaction leads to diminished formation of EGFR dimers on the cell surface, while EGFR is strongly ubiquitinylated. The tyrosine kinase inhibitor GEF has been reported to induce dimer formation by enhancing the affinity of EGF-EGFR, while lapatinib stabilizes the inactive form of EGFR and reduces the affinity of EGF-EGFR interaction, having no effect in dimer formation [26]. Ubiquitin-tagged EGFR is internalized and trafficked through the endocytic pathway, accompanied by other ubiquitin-tagged intracellular effectors, ultimately targeting the receptor for lysosomal degradation, thereby ensuring termination of the signal [43]. Bearing these facts in mind, CSMD1 seems to interfere with the formation of EGFR dimers and directs Ub-EGFR in general to a rapid endosomal trafficking. Of note, this rapid endosomal trafficking does not seem to be driven by EGF-EGFR affinity and internalization rate, as both these aspects were similar in CSMD1-expressing and CTRL cells. The CSMD1-EGFR interaction in turn, results in a decreased EGFR intracellular kinase domain phosphorylation, and subsequent interference with the PI3K-AKT signaling cascade downstream of EGFR. A recent study in head and neck squamous cell carcinomas highlighted that genomic aberrations including *EGFR* amplifications, *AKT1* amplifications and *CSMD1* deletions, but not *PIK3CA*, were highly associated with responsiveness to PI3K-targeted drugs [44]. In our experimental setup in three different TNBC cell lines, EGF-EGFR induced AKT activation was abrogated in the presence of CSMD1, even in BT-20 BCCs that harbors an activating mutation in *PIK3CA* [45]. However, in several clinical trials, *PIK3CA* mutations are considered as a treatment predictive biomarker and mutation status is an inclusion criterion [46].

Upon EGFR activation, cell surface active receptors are internalized to the early endosomes, where they are sorted either for degradation to terminate the signal, or recycled back to the cell surface to sustain the signal [47]. Intracellular protein degradation occurs most commonly through two major mechanisms, lysosomal degradation and proteasome mediated degradation. Lysosomal degradation occurs through proteolytic enzyme activity within lysosomes while proteasomal degradation is targeted through the ubiquitination of the specific protein. In the case of EGFR, convincing evidence has been reported for ubiquitin-dependent targeting of EGFR to lysosomal degradation. Even though EGFR as such is not a proteasomal target, it has been reported that ubiquitination and proteasomal activity are required for its lysosomal sorting [48]. Our results revealed that CSMD1 directed EGFR preferably to the lysosomal degradation pathway, rather than the proteasomal system. In the presence of CSMD1, EGFR localized faster to the endosomal compartments, while CSMD1 was more strongly associated with the late endosomal marker LAMP1, consistent with the faster degradation rate of EGFR in CSMD1-expressing BCCs.

The main treatments for breast cancer include surgery, chemotherapy, radiotherapy, hormonal therapy as well as targeted cancer drugs. Chemotherapy is introduced to breast cancer treatment as neoadjuvant chemotherapy aiming to shrink locally advanced tumors before surgery, as adjuvant chemotherapy after surgical intervention to eliminate residual cancer cells, as well as a treatment option for advanced metastatic breast cancers [49]. Moreover, inhibition of EGFR signaling is associated with decreased drug efflux activity [50]. In the case of TNBC, which does not respond to hormonal and targeted therapy, the main line of treatment is chemotherapy. In addition, a recent large-scale pharmacogenomics study reported co-occurring resistance between EGFR-RTK inhibitors and chemotherapy in breast cancer [51]. Therefore, it is of significant interest that expression of CSMD1 renders TNBC BCCs more sensitive to chemotherapy in both 2D and 3D cell cultures. In addition, CSMD1 expression increases the effectiveness of combination therapies with first line tyrosine kinase inhibitors and second line treatment chemotherapy at low doses, versus monotherapy treatment. The current study is limited by the lack of in vivo xenografts supporting the interplay between CSMD1 and EGFR axis. These data, albeit suggested by the reviewers, could not be generated due to strict restrictions imposed by Lund University on animal experimentation during Covid pandemic at the period of the manuscript revisions. However, our in vitro observations were further supported by the clinical data from a real-world RNA sequenced cohort, SCAN-B, where *CSDM1* expression showed a trend towards better responses of TNBC patients to adjuvant chemotherapy treatment, while *EGFR* expression further characterize that response.

## Conclusions

**Fig. 8:**
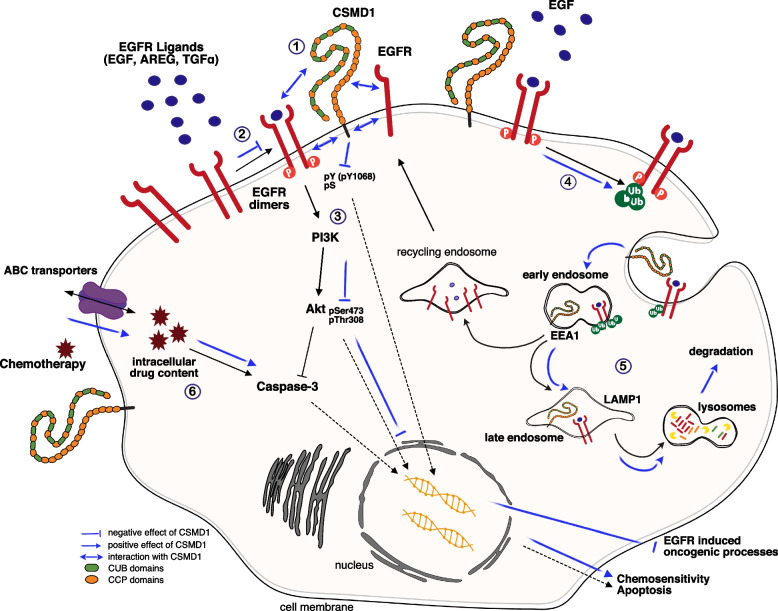
Overview of CSMD1 proposed mechanism of action. (1) CSMD1 strongly interacted with either monomeric or dimeric EGFR both in the extra- and intracellular domains. This interaction led to (2) diminished EGFR dimers formation and (3) EGFR signaling (decreased EGFR intracellular kinase domain phosphorylation and PI3K-AKT signaling cascade activation). (4) In the presence of CSMD1, EGFR was highly ubiquitinylated, thus exhibited altered endosomal trafficking. (5) CSMD1 enhanced EGFR degradation, while EGFR was faster localized to early (EEA1) and late (LAMP1) endosomes. (6) CSMD1-expressing BBCs were more sensitive upon chemotherapy treatment, demonstrating lower drug efflux activity and increased levels of cleaved caspase-3

In conclusion, we have characterized one novel mechanism of tumor suppressor activity of CSMD1 and we have identified EGFR as the mediator of this action (Fig. 8). This study highlights the possibility that CSMD1 may be used as a biomarker predicting chemotherapy response in TNBC, which should be also investigated for other subtypes. Thus, CSMD1 expression may help to guide treatment options, aiding a personalized treatment plan. Furthermore, if CSMD1 expression can be upregulated in tumors in vivo, CSMD1 may also be considered as a potential avenue for a novel therapy.

## Supplementary Information

**Additional file 1**: **S.Figure 1** Expression of mRNA coding for (A) *EGF*, (B) *TGF-α* and (C) *AREG* in MDA-MB-231 CTRL and CSMD1 clonal cells. *EGFR* gene expression (FPKM) plotted against *CSMD1* (FPKM) gene expression in (D) all BC patients and in (E) TNBC patients of SCAN-B cohort (F-G) Protein extracts of MDA-MB-231 BCCs were immunoprecipitated with anti-EGFR. Eluted proteins were analyzed by immunoblotting with (F) anti-phosphotyrosine (pTyr) or anti-EGFR antibody and (G) anti-phosphoserine (pSer) or anti-EGFR antibody, as indicated. (H & I) Densitometric western blot analysis of total phosphorylation tyrosine and serine residues of EGFR. Bars display mean ± SD. Mann–Whitney comparison test was used (*<0.05). (J) Binding assays with ^125^I-labeled EGF in CTRL and CSMD1 MDA-MB-231 BCCs. All experiments were repeated at least 3 times with bars indicating mean ± SD, grey circles correspond to independent data points for CTRL and CSMD1 groups, respectively. **S.Figure 2**. (A) Ubiquitinated EGFR was examined via EGFR immunoprecipitation followed by immunoblotting with anti-ubiquitin antibody in denaturing lysates. Representative blots from three independent experiments are presented in CTRL and CSMD1 MDA-MB-231 BCCs. (B) EGFR internalization kinetics using ^125^I-EGF in MDA-MB-231 BCCs. The amounts of internalized and surface 125I-EGF (cpm) where plotted against time upper panel, while the ratio of internalized/surface EGF against time was used to calculate the internalization rate constant ke. (C) Fractionation analysis in cytosol and membrane of CTRL and CSMD1 MDA-MB-231 BCCs upon stimulation with EGF (25 ng/mL) for 2h. Representative blots are shown. The fractions were blotted for CSMD1, EGFR, EEA1, LAMP1, β-tubulin and NA/K ATPase (D) Ratio of cytosolic to membrane EGFR was calculated. Bars display mean ± SD. **S. Figure 3** Validation of the major findings in BT-20 TNBC cell line (A) Cell lysates were immunoprecipitated using antibodies against CSMD1 or corresponding IgG control followed by immunodetection of EGFR and CSMD1 in BT-20 cells. (B) Immunoblot analysis of phosphorylated EGFR at the residue Y1068, total EGFR and β-tubulin used as a loading control. (C) Densitometry of pEGFR Y1068 normalized to total EGFR. (D) Densitometry of pEGFR Y1068 normalized to β-tubulin, and (E) densitometry of total EGFR normalized to β-tubulin. (F) Immunoblot analysis of phosphorylated Akt at the residue Ser473 (pAkt Ser473), total Akt and GAPDH used as a loading control in BT-20 cells. (G) Densitometry of pAkt Ser473 normalized to total Akt and (H) densitometry of pAkt Ser473 normalized to GAPDH in BT-20 cells. A two-way ANOVA Bonferroni’s multiple comparisons test was used when comparing 3 or more groups with 2 variables (*<0.05). (I) EGFR degradation: representative immunoblots of EGFR levels in lysates of BT-20 CTRL and CSMD1 overexpressing cells, pre-treated with translation inhibitor cycloheximide (100 g/mL) for 2 h, followed by EGF stimulation (25 ng/ml) for 0, 2, 4, 8 hours (h). (J) Quantification of EGFR levels in MDA-MB-231 cells plotted against time. A two-way ANOVA Bonferroni’s multiple comparisons test was used when comparing CTRL and CSMD1 groups (*<0.05, **<0.01). (K) BT-20 CTRL and CSMD1 cells were treated with different chemotherapy agents (doxorubicin and epirubicin) for 48h, and apoptosis was monitored using annexin V staining while live/dead cell discrimination was performed with Zombie Aqua staining, both using flow cytometry. Bar graphs showing percentages of late apoptotic cells. All experiments were repeated at least 3 times with bars indicating mean ± SD, grey circles correspond to independent data points for CTRL and CSMD1 groups, respectively. A two-way ANOVA Bonferroni’s multiple comparisons test was used when comparing CTRL and CSMD1 groups in different concentration of chemotherapy drugs or treatments. **S. Figure 4** Validation of the major findings in MDA-MB-468 TNBC cell line (A) Immunoblot analysis of phosphorylated EGFR at the residue Y1068, total EGFR and β-tubulin in MDA-MB-468 cells. (B) Densitometry of pEGFR-Y1068 normalized to total EGFR. (C) Densitometry of pEGFR-Y1068 normalized to β-tubulin, and (D) densitometry of total EGFR normalized to β-tubulin. (E) Immunoblot analysis of phosphorylated Akt at the residue Ser473 (pAkt-Ser473), total Akt and GAPDH in MDA-MB-468 cells. (F) Densitometry of pAkt-Ser473 normalized to total Akt and (G) densitometry of pAkt-Ser473 normalized to GAPDH. A two-way ANOVA Bonferroni’s multiple comparisons test was used when comparing 3 or more groups with 2 variables (*<0.05). (H) EGFR degradation in MDA-MB-468 breast cancer cell line: detection of EGFR in lysates of MDA-MB-468 CTRL and CSMD1-overexpressing cells, pre-treated with translation inhibitor cycloheximide (100 g/mL) for 2 h followed by EGF stimulation (25 ng/ml) for 0, 2, 4, 8 hours (h). (I) Quantification of EGFR levels in MDA-MB-468 cells plotted against time. A two-way ANOVA Bonferroni’s multiple comparisons test was used when comparing CTRL and CSMD1 groups (*<0.05, **<0.01). (J) MDA-MB-468 CTRL and CSMD1-overexpressing cells were treated with different chemotherapy agents (doxorubicin and epirubicin) for 48h, and apoptosis was monitored using annexin V staining while live/dead cell discrimination was performed with Zombie Aqua staining, both using flow cytometry. All experiments were repeated at least 3 times with bars indicating mean ± SD, grey circles correspond to independent data points for CTRL and CSMD1 groups, respectively. Bar graphs show percentages of late apoptotic cells. A two-way ANOVA Bonferroni’s multiple comparisons test was used when comparing CTRL and CSMD1 groups in different concentration of chemotherapy drugs or treatments. **S. Figure 5** (A) Drug efflux activity in MDA-MB-231 CSMD1-overexpressing cells and CTRL cells. Representative histograms are shown. (B) Bar graphs showing gMFI of intracellular doxorubicin content in MDA-MB-231 CSMD1 and CTRL cells. Student’s t-test was used when comparing CTRL and CSMD1 (C) Representative immunoblots of autophagy markers p62 and LC3B in lysates of MDA-MB-231 CTRL and CSMD1 cells upon treatment with GEF and CQ and combination of them for 24h. All experiments were repeated at least 3 times with bars indicating mean ± SD, grey circles correspond to independent data points for CTRL and CSMD1 groups, respectively. **S. Figure 6** Frequency (%) of *CSMD1* expression in (A) all BC patients and in (B) TNBC patients of SCAN-B cohort. (C) Box plot of *EGFR* gene expression for tumor samples of SCAN-B cohort subset of TNBC stratified according to basal and non-basal like properties. **Table S1** List of antibodies used in this study.

## Data Availability

The datasets during and/or analyzed during the current study available from the corresponding author on reasonable request.

## Abbreviations

AREG: Amphiregulin
BCCs: Breast cancer cells
CCP: Complement control protein
CHX: Cycloheximide
CSMD1: CUB and Sushi multiple domains 1
CTSS: Cathepsin S
EEA1: Early endosome antigen 1
EGFR: Epidermal growth factor receptor
ER: Estrogen receptor
FBS: Fetal bovine serum
GEF: Gefitinib
HER2: Human epidermal growth factor receptor 2
LAMP1: Lysosomal-associated membrane protein 1
LN: Lymph node
NEM: N-Ethylmaleimide
PLA: Proximity ligation assay
TNBC: Triple negative breast cancer
Ub: Ubiquitin
