# Correction: Complement inhibitor CSMD1 modulates epidermal growth factor receptor oncogenic signaling and sensitizes breast cancer cells to chemotherapy

**DOI:** 10.1186/s13046-023-02703-3

**Published:** 2023-05-30

**Authors:** Chrysostomi Gialeli, Emre Can Tuysuz, Johan Staaf, Safa Guleed, Veronika Paciorek, Matthias Mörgelin, Konstantinos S. Papadakos, Anna M. Blom

**Affiliations:** 1 Department of Translational Medicine, Lund University, Malmö, Sweden; 2 Experimental Cardiovascular Research Group, Department of Clinical Sciences, Lund University, Malmö, Sweden; 3 Division of Oncology, Department of Clinical Sciences Lund, Lund University, Medicon Village, Lund, Sweden; 4 Colzyx AB, Lund, Sweden

**Correction: *J Exp Clin Cancer Res*** 40, 258 (2021)

**https://doi.org/10.1186/s13046-021-02042-1**

Following publication of the original article [1], an error was identified Fig. 1, specifically:

- Fig. 1a and b—The image of wild type MDA-MB-231 cells was inadvertently duplicated.

The correct Fig. 1 is presented below:

**Fig. 1:**
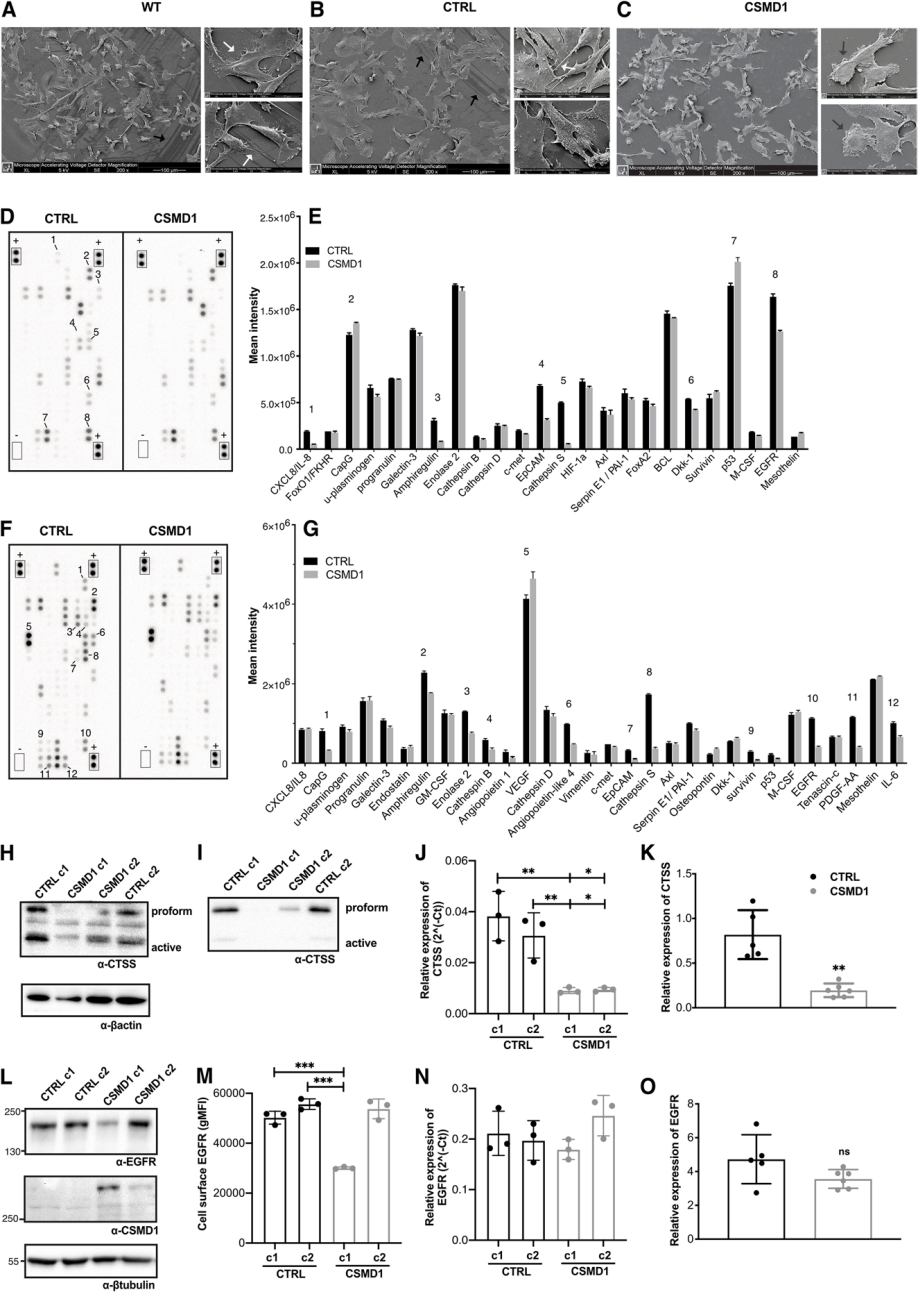
Distinct proteomic signature of CSMD1-expressing MDA-MB-231 BCCs. Scanning electron microscope images of MDA-MB-231 **A** WT, **B** CTRL and **C** CSMD1 BCCs showing distinct morphology of CSMD1-expressing cells. Large panels scale 100 μm. Small panels scale 20 μm. Black arrows indicate the cell “footprints”. White arrows indicate the cytoplasmic protrusions. Dark grey arrows indicate the extracellular material-globular vesicles. Proteome Oncology profiler array—Cancer-related protein analysis of CTRL and CSMD1-expressing MDA-MB-231 cells. **D, E** Blots showing the location of proteins and capture antibodies spotted onto the array in duplicates. Positive and negative controls are indicated by + and – adjacent to appropriate spots. Quantification of mean spot pixel intensities of CTRL and CSMD1 cells was plotted in the same order as spotted in the array when analyzing (**D**) cell lysates with the (**E**) corresponding quantifications and (**F**) cell culture supernatants with the (**G**) corresponding quantifications. Numbers correspond to interesting findings in this array. Confirmation of the array: CTSS and EGFR expression in different CTRL and CSMD1 clones of MDA-MB-231 BCCs. Western blot analysis of total cell **H** lysates and **I** supernatants immunodetection of CTSS with β-actin used as a loading control, and **J** mRNA expression levels of *CTSS*. **L** Western blot analysis of total cell lysates immunodetecting EGFR and CSMD1 with β-tubulin used as a loading control, **M** cell surface *EGFR* expression assessed by flow cytometry, and **N** mRNA expression levels of EGFR. Shown is also mRNA expression of **K** *CTSS* and **O** *EGFR* in tumors formed in SCID mice injected with MDA-MB-231 CTRL and CSMD1 cells (5 mice in each group). All experiments were repeated at least 3 times. Bars indicate means ± SD. One-way ANOVA Turkey’s multiple comparisons test was used when comparing CSMD1 clones to CTRL clones, and Mann–Whitney comparison test was used when comparing CTRL and CSMD1 groups in tumors formed in vivo (* < 0.05, ** < 0.01, * < 0.001)

The correction does not affect the overall Conclusion of the article.
