# Supplementary material for: Complement inhibitor CSMD1 modulates epidermal growth factor receptor oncogenic signaling and sensitizes breast cancer cells to chemotherapy
Source: J Exp Clin Cancer Res. 2021 Aug 17;40:258. doi: 10.1186/s13046-021-02042-1 (PMC8371905; doi:10.1186/s13046-021-02042-1)
Supplement: Supplementary file 1 — Additional file 1: S.Figure 1 Expression of mRNA coding for (A) EGF, (B) TGF-α and (C) AREG in MDA-MB-231 CTRL and CSMD1 clonal cells. EGFR gene expression (FPKM) plotted against CSMD1 (FPKM) gene expression in (D) all BC patients and in (E) TNBC patients of SCAN-B cohort (F-G) Protein extracts of MDA-MB-231 BCCs were immunoprecipitated with anti-EGFR. Eluted proteins were analyzed by immunoblotting with (F) anti-phosphotyrosine (pTyr) or anti-EGFR antibody and (G) anti-phosphoserine (pSer) or anti-EGFR antibody, as indicated. (H & I) Densitometric western blot analysis of total phosphorylation tyrosine and serine residues of EGFR. Bars display mean ± SD. Mann–Whitney comparison test was used (*<0.05). (J) Binding assays with 125I-labeled EGF in CTRL and CSMD1 MDA-MB-231 BCCs. All experiments were repeated at least 3 times with bars indicating mean ± SD, grey circles correspond to independent data points for CTRL and CSMD1 groups, respectively. S.Figure 2. (A) Ubiquitinated EGFR was examined via EGFR immunoprecipitation followed by immunoblotting with anti-ubiquitin antibody in denaturing lysates. Representative blots from three independent experiments are presented in CTRL and CSMD1 MDA-MB-231 BCCs. (B) EGFR internalization kinetics using 125I-EGF in MDA-MB-231 BCCs. The amounts of internalized and surface 125I-EGF (cpm) where plotted against time upper panel, while the ratio of internalized/surface EGF against time was used to calculate the internalization rate constant ke. (C) Fractionation analysis in cytosol and membrane of CTRL and CSMD1 MDA-MB-231 BCCs upon stimulation with EGF (25 ng/mL) for 2h. Representative blots are shown. The fractions were blotted for CSMD1, EGFR, EEA1, LAMP1, β-tubulin and NA/K ATPase (D) Ratio of cytosolic to membrane EGFR was calculated. Bars display mean ± SD. S. Figure 3 Validation of the major findings in BT-20 TNBC cell line (A) Cell lysates were immunoprecipitated using antibodies against CSMD1 or corresponding IgG control foll [file 13046_2021_2042_MOESM1_ESM.zip › Supplementary materials and methods .docx]

**Binding of ^125^I-EGF**

Cells were incubated with increasing concentrations of ^125^I-EGF (Perkin Elmer) on ice for 30min. The cells were washed three times with ice cold PBS before surface-bound ^125^I-EGF was removed by incubating the cells with acetic buffer (0.2 M acetic acid, 0.5 M NaCl, pH 2.5) for 5 minutes on ice followed by a wash with the same buffer (surface). The radioactivity released from the cell surface was subsequently measured in a γ-counter. Thereafter, the cells were hydrolyzed with 1 M NaOH on ice for 30 minutes and the internalized ^125^I-EGF was measured in a γ-counter (internalized). Unlabeled EGF (100 M excess) was added to along with ^125^I-EGF to measure the non-specific binding, which in all cases was less than 5% and was taken into account in the calculations.

**Internalization of ^125^I-EGF**

Cells were incubated with 7 nM of ^125^I-EGF (Perkin Elmer) for 1, 2, 3, 4, 5, 6 and 7 minutes at 37^o^C. The cells were washed three times with ice cold PBS before surface-bound ^125^I-EGF was removed by incubating the cells with acetic buffer (0.2 M acetic acid, 0.5 M NaCl, pH 2.5) for 5 minutes on ice followed by a wash with the same buffer (surface). The radioactivity released from the cell surface was subsequently measured in a γ-counter. Thereafter, the cells were hydrolyzed with 1 M NaOH on ice for 30 minutes and the internalized ^125^I-EGF was measured in a γ-counter (internalized). Unlabeled EGF (100 M excess) was added to along with ^125^I-EGF to measure the non-specific binding, which in all cases was less than 5% and was taken into account in the calculations. The specific internalization rate constant k_e_ was calculated by plotting the ratio of internalized to surface EGF through time, k_e_ corresponds to the linear regression coefficient [1].

**Subcellular fractionation**

Cytoplasmic and membrane extracts were prepared according to the instructions of the Mem-PER^TM^ Plus Membrane protein extraction kit (Pierce).

**Doxorubicin Efflux Assay**

Cells were seeded on 6-well plate and incubated overnight. Next day, cells were pre-treated with 4 μM Doxorubucin for 4 hours. After the pre-treatment, cells were washed with PBS, drug-free medium was added on cells and incubated at 37^o^C for 40 hours. Cells were then de-attached with trypsin and washed with PBS for 2 times. To detect cellular intensity of doxorubicin, doxorubicin was excited with 488 nm laser and emitted light was collected with a 585/42 nm filter analyzed using Cytoflex flow cytometer (Beckman) and FlowJo software.

**References**

[1] H.S. Wiley, D.D. Cunningham, The endocytotic rate constant. A cellular parameter for quantitating receptor-mediated endocytosis, The Journal of biological chemistry 257(8) (1982) 4222-9.
