# Supplementary material for: Complement inhibitor CSMD1 modulates epidermal growth factor receptor oncogenic signaling and sensitizes breast cancer cells to chemotherapy
Source: J Exp Clin Cancer Res. 2021 Aug 17;40:258. doi: 10.1186/s13046-021-02042-1 (PMC8371905; doi:10.1186/s13046-021-02042-1)
Supplement: Supplementary file 1 — Additional file 1: S.Figure 1 Expression of mRNA coding for (A) EGF, (B) TGF-α and (C) AREG in MDA-MB-231 CTRL and CSMD1 clonal cells. EGFR gene expression (FPKM) plotted against CSMD1 (FPKM) gene expression in (D) all BC patients and in (E) TNBC patients of SCAN-B cohort (F-G) Protein extracts of MDA-MB-231 BCCs were immunoprecipitated with anti-EGFR. Eluted proteins were analyzed by immunoblotting with (F) anti-phosphotyrosine (pTyr) or anti-EGFR antibody and (G) anti-phosphoserine (pSer) or anti-EGFR antibody, as indicated. (H & I) Densitometric western blot analysis of total phosphorylation tyrosine and serine residues of EGFR. Bars display mean ± SD. Mann–Whitney comparison test was used (*<0.05). (J) Binding assays with 125I-labeled EGF in CTRL and CSMD1 MDA-MB-231 BCCs. All experiments were repeated at least 3 times with bars indicating mean ± SD, grey circles correspond to independent data points for CTRL and CSMD1 groups, respectively. S.Figure 2. (A) Ubiquitinated EGFR was examined via EGFR immunoprecipitation followed by immunoblotting with anti-ubiquitin antibody in denaturing lysates. Representative blots from three independent experiments are presented in CTRL and CSMD1 MDA-MB-231 BCCs. (B) EGFR internalization kinetics using 125I-EGF in MDA-MB-231 BCCs. The amounts of internalized and surface 125I-EGF (cpm) where plotted against time upper panel, while the ratio of internalized/surface EGF against time was used to calculate the internalization rate constant ke. (C) Fractionation analysis in cytosol and membrane of CTRL and CSMD1 MDA-MB-231 BCCs upon stimulation with EGF (25 ng/mL) for 2h. Representative blots are shown. The fractions were blotted for CSMD1, EGFR, EEA1, LAMP1, β-tubulin and NA/K ATPase (D) Ratio of cytosolic to membrane EGFR was calculated. Bars display mean ± SD. S. Figure 3 Validation of the major findings in BT-20 TNBC cell line (A) Cell lysates were immunoprecipitated using antibodies against CSMD1 or corresponding IgG control foll [file 13046_2021_2042_MOESM1_ESM.zip › Table S1.docx]

**Table S1**: List of antibodies used in this study

| ANTIBODY | COMPANY | CATALOGUE No |
| --- | --- | --- |
| Anti-AKT | Cell Sign. Tech. | 4691 |
| Anti-c-Myc Tag | Thermo Fish. Sc. | MA1-21316 |
| Anti-cleaved CASP3 | Cell Sign. Tech. | 9664 |
| Anti-CSMD1 | Custom made - Agrisera | Polyclonal rabbit |
| Anti-CTSS | R&D systems | AF1183 |
| Anti-EEA1 | Cell Sign. Tech. | 48453 |
| Anti-EGFR | R&D systems | AF231 |
| Anti-EGFR | Cell Sign. Tech. | 4267 |
| Anti-EGFR Brilliant Violet 421 | BioLegend | 332911 |
| Anti-GAPDH | Abcam | ab8245 |
| Anti-Goat IgG | R&D systems | AB-108-C |
| Anti-LAMP1 | Cell Sign. Tech. | 15665 |
| Anti-LC3B | Cell Sign. Tech. | 2775 |
| Anti-Mouse IgG1, κ Isotype Brilliant Violet 421 | BioLegend | 400157 |
| Anti-Na/K ATPase | Abcam | ab76020 |
| Anti-p62 | Abcam | ab56416 |
| Anti-pAKT (Ser473) | Cell Sign. Tech. | 4060 |
| Anti-pAKT (Thr308) | Cell Sign. Tech. | 13038 |
| Anti-pEGFR Y1068 | R&D systems | ΜΑΒ8967 |
| Anti-phosphoserine | Abcam | ab9332 |
| Anti-phosphotyrosine | Merk Millipore | 05-321 |
| Anti-Rabit IgG | Custom made - Agrisera | Polyclonal rabbit |
| Anti-Ub | Cell Sign. Tech. | 3936 |
| Anti-β-actin | Abcam | ab8226 |
| Anti-β-tubulin | Abcam | ab6046 |
| Donkey anti-Goat IgG/ Alx647 | Thermo Fish. Sc. | A32849 |
| Goat anti-Mouse IgG Alx405 | Thermo Fish. Sc. | A31553 |
| Goat anti-Rabbit IgG Alx488 | Thermo Fish. Sc. | A11034 |
| Polyclonal Goat Anti-Mouse IgG/HRP | DAKO | P0447 |
| Polyclonal Goat Anti-Rabbit Ig/HRP | DAKO | P0448 |
| Polyclonal Goat Anti-rabbit IgG/HRP | Cell Sign. Tech. | 7074 |
| Polyclonal Rabbit Anti-Goat IgG/HRP | DAKO | P0449 |
| Rabbit anti-Goat IgG Alx647 | Thermo Fish. Sc. | A21446 |
