# Supplementary figures and images for: Complement inhibitor CSMD1 modulates epidermal growth factor receptor oncogenic signaling and sensitizes breast cancer cells to chemotherapy
Source: J Exp Clin Cancer Res. 2021 Aug 17;40:258. doi: 10.1186/s13046-021-02042-1 (PMC8371905; doi:10.1186/s13046-021-02042-1)

Supplementary Figure 1

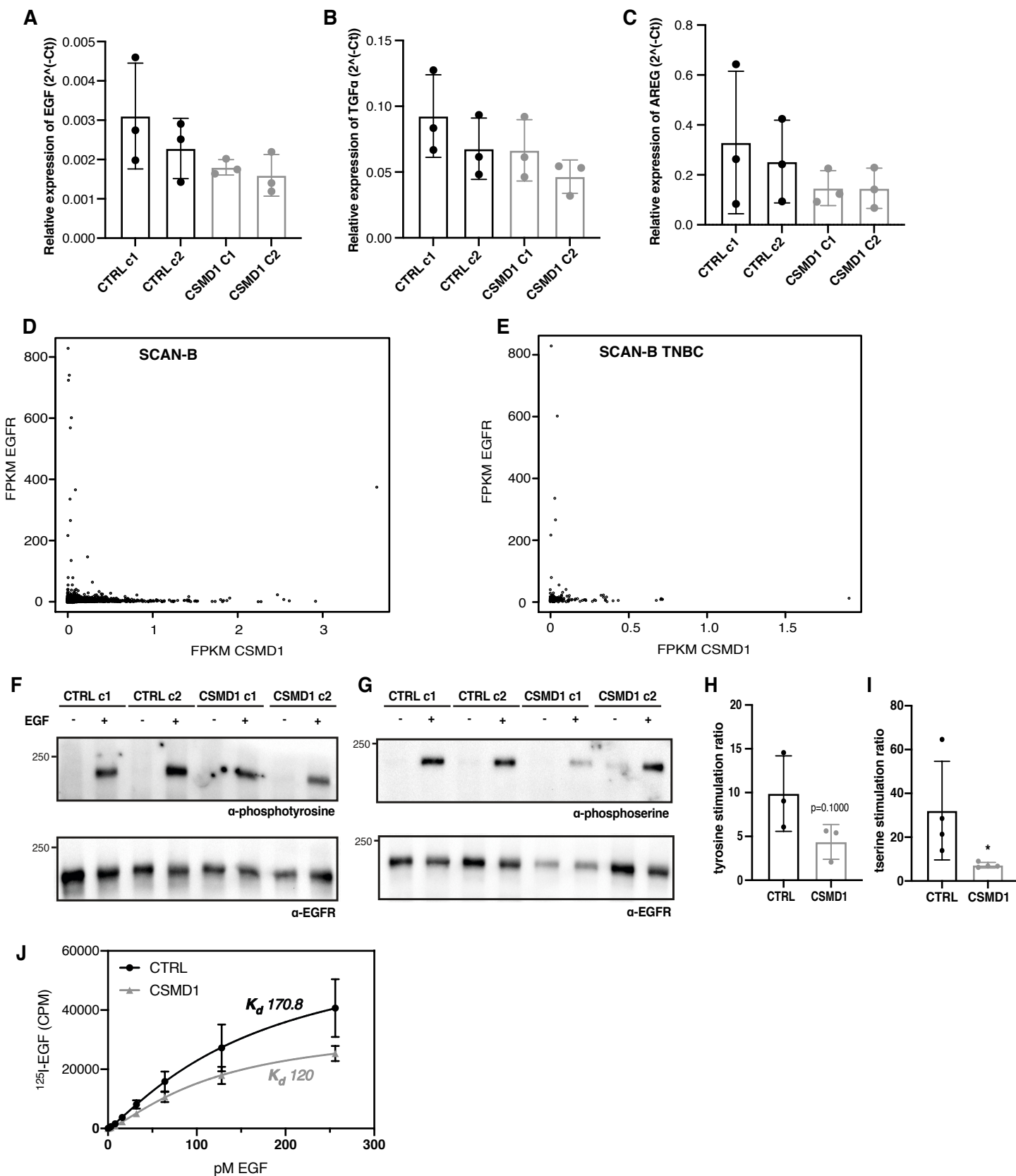

Supplement: Supplementary file 1 — Additional file 1: S.Figure 1 Expression of mRNA coding for (A) EGF, (B) TGF-α and (C) AREG in MDA-MB-231 CTRL and CSMD1 clonal cells. EGFR gene expression (FPKM) plotted against CSMD1 (FPKM) gene expression in (D) all BC patients and in (E) TNBC patients of SCAN-B cohort (F-G) Protein extracts of MDA-MB-231 BCCs were immunoprecipitated with anti-EGFR. Eluted proteins were analyzed by immunoblotting with (F) anti-phosphotyrosine (pTyr) or anti-EGFR antibody and (G) anti-phosphoserine (pSer) or anti-EGFR antibody, as indicated. (H & I) Densitometric western blot analysis of total phosphorylation tyrosine and serine residues of EGFR. Bars display mean ± SD. Mann–Whitney comparison test was used (*<0.05). (J) Binding assays with 125I-labeled EGF in CTRL and CSMD1 MDA-MB-231 BCCs. All experiments were repeated at least 3 times with bars indicating mean ± SD, grey circles correspond to independent data points for CTRL and CSMD1 groups, respectively. S.Figure 2. (A) Ubiquitinated EGFR was examined via EGFR immunoprecipitation followed by immunoblotting with anti-ubiquitin antibody in denaturing lysates. Representative blots from three independent experiments are presented in CTRL and CSMD1 MDA-MB-231 BCCs. (B) EGFR internalization kinetics using 125I-EGF in MDA-MB-231 BCCs. The amounts of internalized and surface 125I-EGF (cpm) where plotted against time upper panel, while the ratio of internalized/surface EGF against time was used to calculate the internalization rate constant ke. (C) Fractionation analysis in cytosol and membrane of CTRL and CSMD1 MDA-MB-231 BCCs upon stimulation with EGF (25 ng/mL) for 2h. Representative blots are shown. The fractions were blotted for CSMD1, EGFR, EEA1, LAMP1, β-tubulin and NA/K ATPase (D) Ratio of cytosolic to membrane EGFR was calculated. Bars display mean ± SD. S. Figure 3 Validation of the major findings in BT-20 TNBC cell line (A) Cell lysates were immunoprecipitated using antibodies against CSMD1 or corresponding IgG control foll [file 13046_2021_2042_MOESM1_ESM.zip › Supplementary Figure 1.pdf]

**Supplementary figure 2**

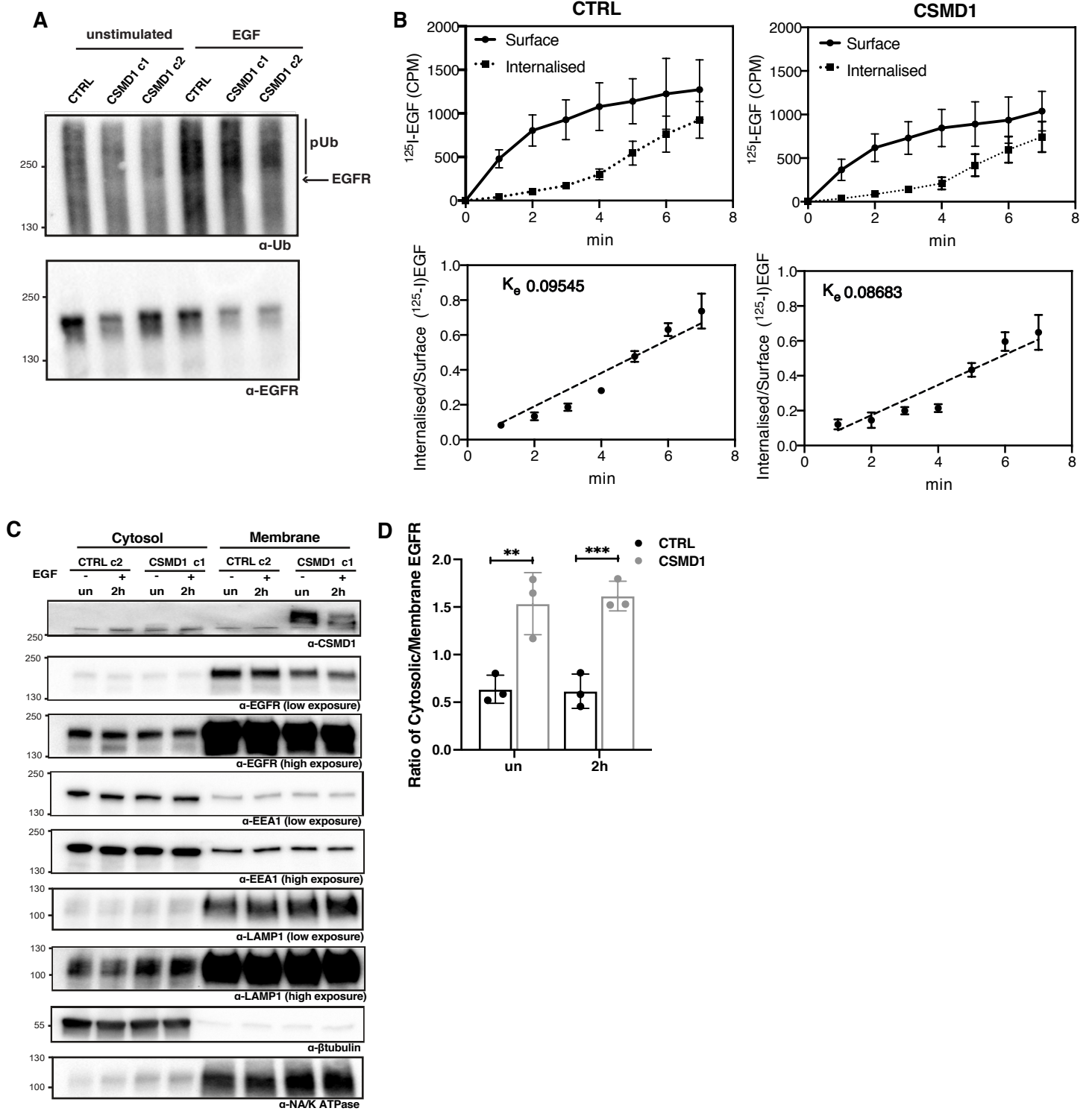

Supplement: Supplementary file 1 — Additional file 1: S.Figure 1 Expression of mRNA coding for (A) EGF, (B) TGF-α and (C) AREG in MDA-MB-231 CTRL and CSMD1 clonal cells. EGFR gene expression (FPKM) plotted against CSMD1 (FPKM) gene expression in (D) all BC patients and in (E) TNBC patients of SCAN-B cohort (F-G) Protein extracts of MDA-MB-231 BCCs were immunoprecipitated with anti-EGFR. Eluted proteins were analyzed by immunoblotting with (F) anti-phosphotyrosine (pTyr) or anti-EGFR antibody and (G) anti-phosphoserine (pSer) or anti-EGFR antibody, as indicated. (H & I) Densitometric western blot analysis of total phosphorylation tyrosine and serine residues of EGFR. Bars display mean ± SD. Mann–Whitney comparison test was used (*<0.05). (J) Binding assays with 125I-labeled EGF in CTRL and CSMD1 MDA-MB-231 BCCs. All experiments were repeated at least 3 times with bars indicating mean ± SD, grey circles correspond to independent data points for CTRL and CSMD1 groups, respectively. S.Figure 2. (A) Ubiquitinated EGFR was examined via EGFR immunoprecipitation followed by immunoblotting with anti-ubiquitin antibody in denaturing lysates. Representative blots from three independent experiments are presented in CTRL and CSMD1 MDA-MB-231 BCCs. (B) EGFR internalization kinetics using 125I-EGF in MDA-MB-231 BCCs. The amounts of internalized and surface 125I-EGF (cpm) where plotted against time upper panel, while the ratio of internalized/surface EGF against time was used to calculate the internalization rate constant ke. (C) Fractionation analysis in cytosol and membrane of CTRL and CSMD1 MDA-MB-231 BCCs upon stimulation with EGF (25 ng/mL) for 2h. Representative blots are shown. The fractions were blotted for CSMD1, EGFR, EEA1, LAMP1, β-tubulin and NA/K ATPase (D) Ratio of cytosolic to membrane EGFR was calculated. Bars display mean ± SD. S. Figure 3 Validation of the major findings in BT-20 TNBC cell line (A) Cell lysates were immunoprecipitated using antibodies against CSMD1 or corresponding IgG control foll [file 13046_2021_2042_MOESM1_ESM.zip › Supplementary Figure 2.pdf]

Supplementary figure 3

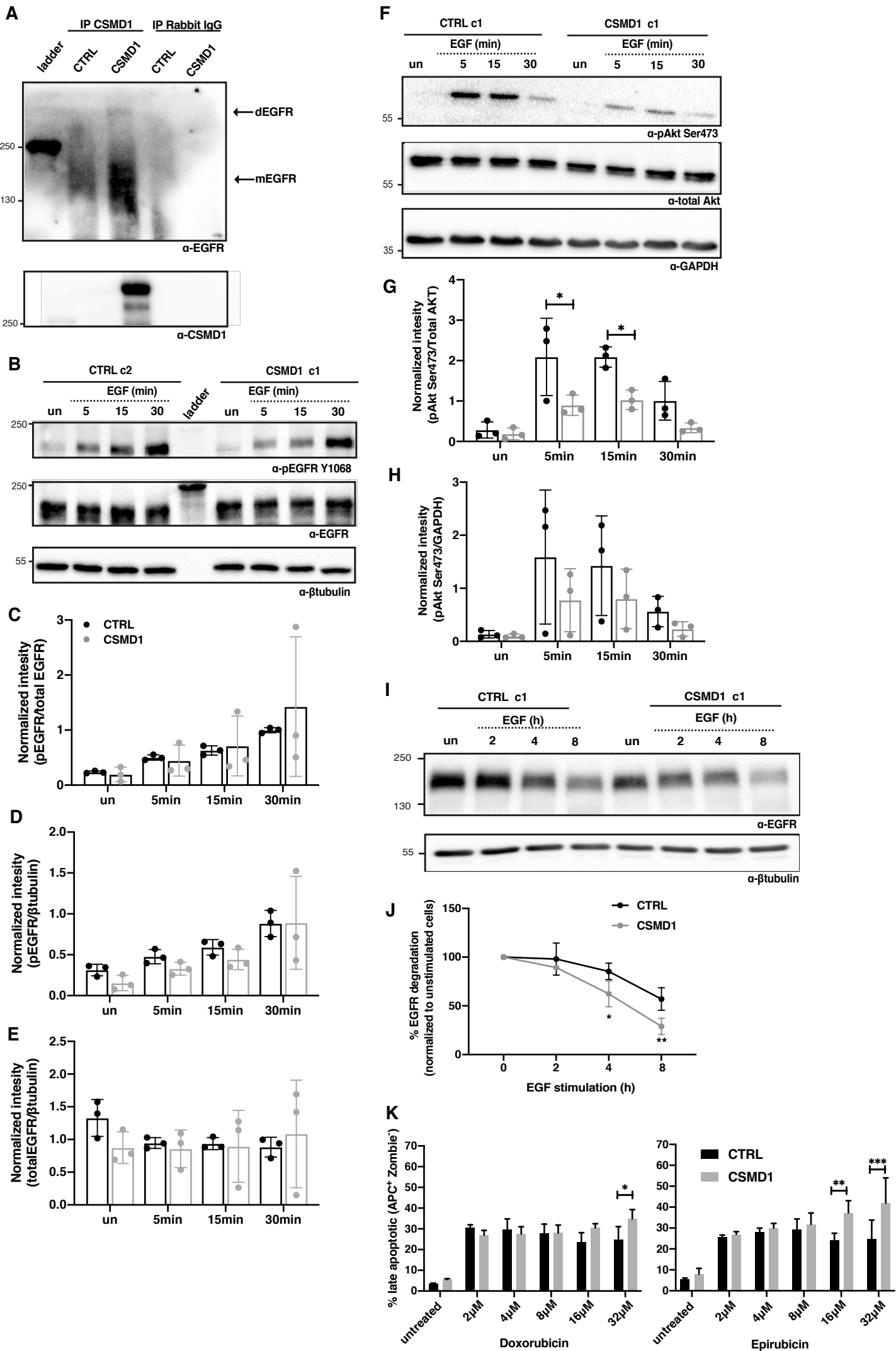

Supplement: Supplementary file 1 — Additional file 1: S.Figure 1 Expression of mRNA coding for (A) EGF, (B) TGF-α and (C) AREG in MDA-MB-231 CTRL and CSMD1 clonal cells. EGFR gene expression (FPKM) plotted against CSMD1 (FPKM) gene expression in (D) all BC patients and in (E) TNBC patients of SCAN-B cohort (F-G) Protein extracts of MDA-MB-231 BCCs were immunoprecipitated with anti-EGFR. Eluted proteins were analyzed by immunoblotting with (F) anti-phosphotyrosine (pTyr) or anti-EGFR antibody and (G) anti-phosphoserine (pSer) or anti-EGFR antibody, as indicated. (H & I) Densitometric western blot analysis of total phosphorylation tyrosine and serine residues of EGFR. Bars display mean ± SD. Mann–Whitney comparison test was used (*<0.05). (J) Binding assays with 125I-labeled EGF in CTRL and CSMD1 MDA-MB-231 BCCs. All experiments were repeated at least 3 times with bars indicating mean ± SD, grey circles correspond to independent data points for CTRL and CSMD1 groups, respectively. S.Figure 2. (A) Ubiquitinated EGFR was examined via EGFR immunoprecipitation followed by immunoblotting with anti-ubiquitin antibody in denaturing lysates. Representative blots from three independent experiments are presented in CTRL and CSMD1 MDA-MB-231 BCCs. (B) EGFR internalization kinetics using 125I-EGF in MDA-MB-231 BCCs. The amounts of internalized and surface 125I-EGF (cpm) where plotted against time upper panel, while the ratio of internalized/surface EGF against time was used to calculate the internalization rate constant ke. (C) Fractionation analysis in cytosol and membrane of CTRL and CSMD1 MDA-MB-231 BCCs upon stimulation with EGF (25 ng/mL) for 2h. Representative blots are shown. The fractions were blotted for CSMD1, EGFR, EEA1, LAMP1, β-tubulin and NA/K ATPase (D) Ratio of cytosolic to membrane EGFR was calculated. Bars display mean ± SD. S. Figure 3 Validation of the major findings in BT-20 TNBC cell line (A) Cell lysates were immunoprecipitated using antibodies against CSMD1 or corresponding IgG control foll [file 13046_2021_2042_MOESM1_ESM.zip › Supplementary Figure 3.pdf]

Supplementary figure 4

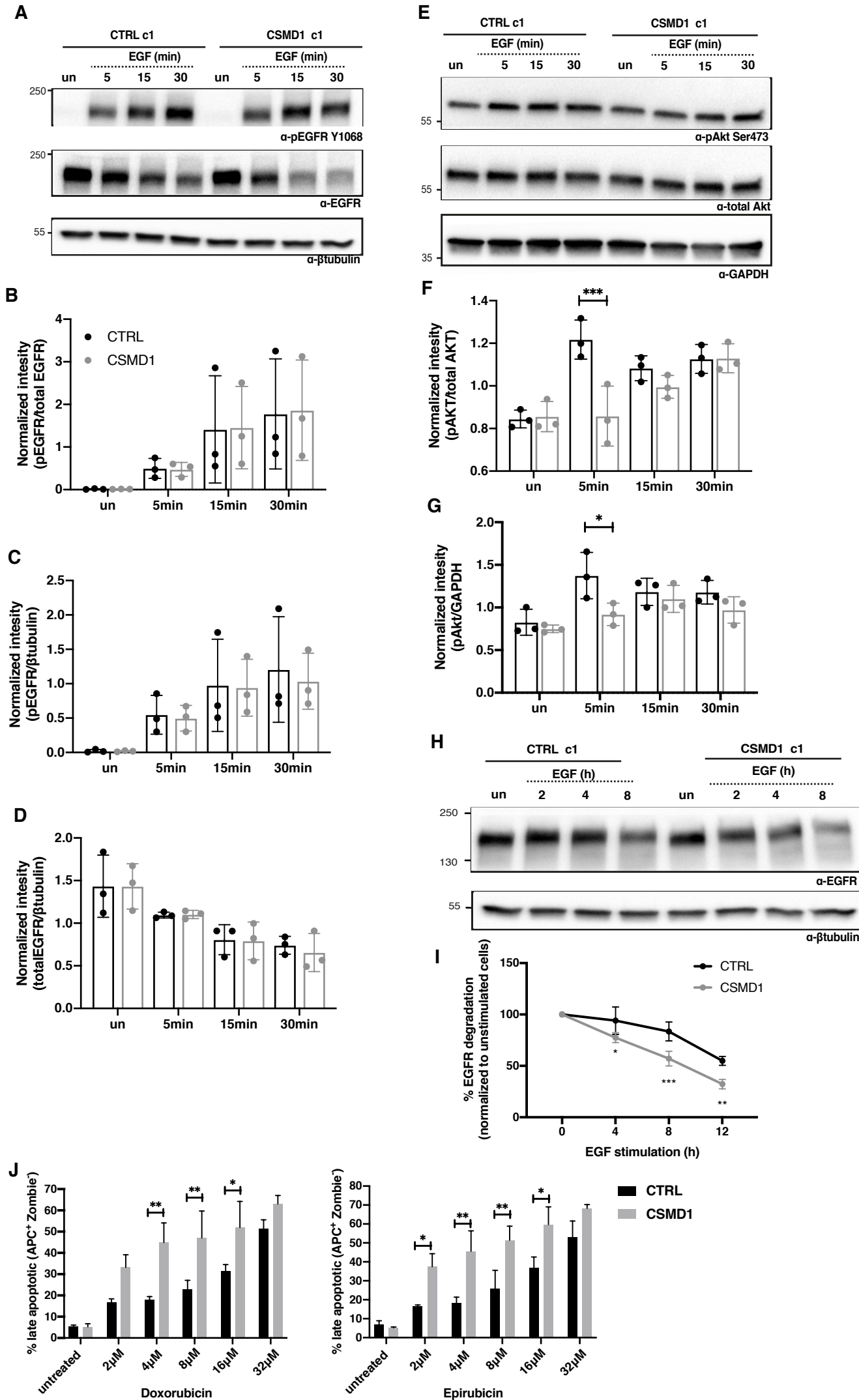

Supplement: Supplementary file 1 — Additional file 1: S.Figure 1 Expression of mRNA coding for (A) EGF, (B) TGF-α and (C) AREG in MDA-MB-231 CTRL and CSMD1 clonal cells. EGFR gene expression (FPKM) plotted against CSMD1 (FPKM) gene expression in (D) all BC patients and in (E) TNBC patients of SCAN-B cohort (F-G) Protein extracts of MDA-MB-231 BCCs were immunoprecipitated with anti-EGFR. Eluted proteins were analyzed by immunoblotting with (F) anti-phosphotyrosine (pTyr) or anti-EGFR antibody and (G) anti-phosphoserine (pSer) or anti-EGFR antibody, as indicated. (H & I) Densitometric western blot analysis of total phosphorylation tyrosine and serine residues of EGFR. Bars display mean ± SD. Mann–Whitney comparison test was used (*<0.05). (J) Binding assays with 125I-labeled EGF in CTRL and CSMD1 MDA-MB-231 BCCs. All experiments were repeated at least 3 times with bars indicating mean ± SD, grey circles correspond to independent data points for CTRL and CSMD1 groups, respectively. S.Figure 2. (A) Ubiquitinated EGFR was examined via EGFR immunoprecipitation followed by immunoblotting with anti-ubiquitin antibody in denaturing lysates. Representative blots from three independent experiments are presented in CTRL and CSMD1 MDA-MB-231 BCCs. (B) EGFR internalization kinetics using 125I-EGF in MDA-MB-231 BCCs. The amounts of internalized and surface 125I-EGF (cpm) where plotted against time upper panel, while the ratio of internalized/surface EGF against time was used to calculate the internalization rate constant ke. (C) Fractionation analysis in cytosol and membrane of CTRL and CSMD1 MDA-MB-231 BCCs upon stimulation with EGF (25 ng/mL) for 2h. Representative blots are shown. The fractions were blotted for CSMD1, EGFR, EEA1, LAMP1, β-tubulin and NA/K ATPase (D) Ratio of cytosolic to membrane EGFR was calculated. Bars display mean ± SD. S. Figure 3 Validation of the major findings in BT-20 TNBC cell line (A) Cell lysates were immunoprecipitated using antibodies against CSMD1 or corresponding IgG control foll [file 13046_2021_2042_MOESM1_ESM.zip › Supplementary Figure 4.pdf]

# Supplementary figure 5

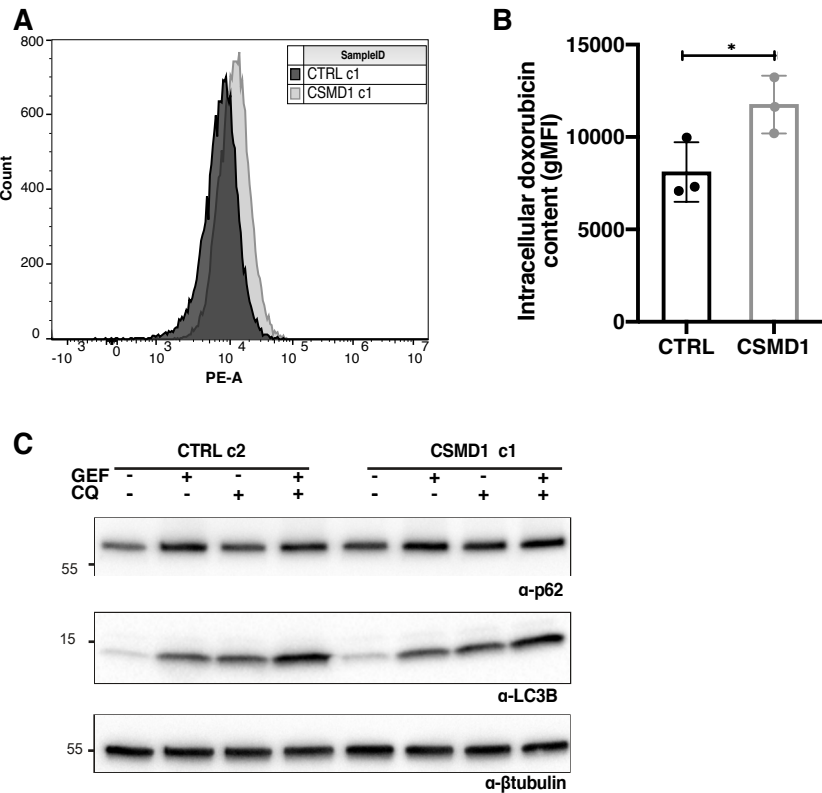

Supplement: Supplementary file 1 — Additional file 1: S.Figure 1 Expression of mRNA coding for (A) EGF, (B) TGF-α and (C) AREG in MDA-MB-231 CTRL and CSMD1 clonal cells. EGFR gene expression (FPKM) plotted against CSMD1 (FPKM) gene expression in (D) all BC patients and in (E) TNBC patients of SCAN-B cohort (F-G) Protein extracts of MDA-MB-231 BCCs were immunoprecipitated with anti-EGFR. Eluted proteins were analyzed by immunoblotting with (F) anti-phosphotyrosine (pTyr) or anti-EGFR antibody and (G) anti-phosphoserine (pSer) or anti-EGFR antibody, as indicated. (H & I) Densitometric western blot analysis of total phosphorylation tyrosine and serine residues of EGFR. Bars display mean ± SD. Mann–Whitney comparison test was used (*<0.05). (J) Binding assays with 125I-labeled EGF in CTRL and CSMD1 MDA-MB-231 BCCs. All experiments were repeated at least 3 times with bars indicating mean ± SD, grey circles correspond to independent data points for CTRL and CSMD1 groups, respectively. S.Figure 2. (A) Ubiquitinated EGFR was examined via EGFR immunoprecipitation followed by immunoblotting with anti-ubiquitin antibody in denaturing lysates. Representative blots from three independent experiments are presented in CTRL and CSMD1 MDA-MB-231 BCCs. (B) EGFR internalization kinetics using 125I-EGF in MDA-MB-231 BCCs. The amounts of internalized and surface 125I-EGF (cpm) where plotted against time upper panel, while the ratio of internalized/surface EGF against time was used to calculate the internalization rate constant ke. (C) Fractionation analysis in cytosol and membrane of CTRL and CSMD1 MDA-MB-231 BCCs upon stimulation with EGF (25 ng/mL) for 2h. Representative blots are shown. The fractions were blotted for CSMD1, EGFR, EEA1, LAMP1, β-tubulin and NA/K ATPase (D) Ratio of cytosolic to membrane EGFR was calculated. Bars display mean ± SD. S. Figure 3 Validation of the major findings in BT-20 TNBC cell line (A) Cell lysates were immunoprecipitated using antibodies against CSMD1 or corresponding IgG control foll [file 13046_2021_2042_MOESM1_ESM.zip › Supplementary Figure 5.pdf]

**Supplementary figure 6**

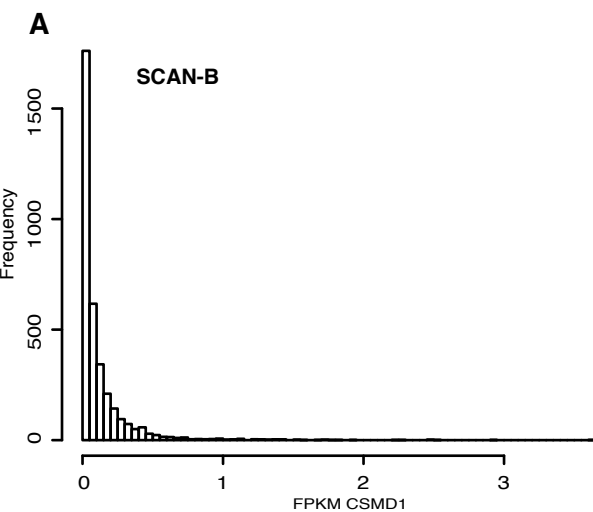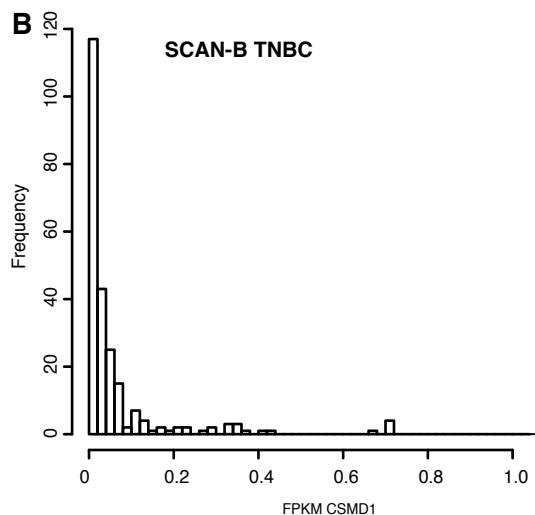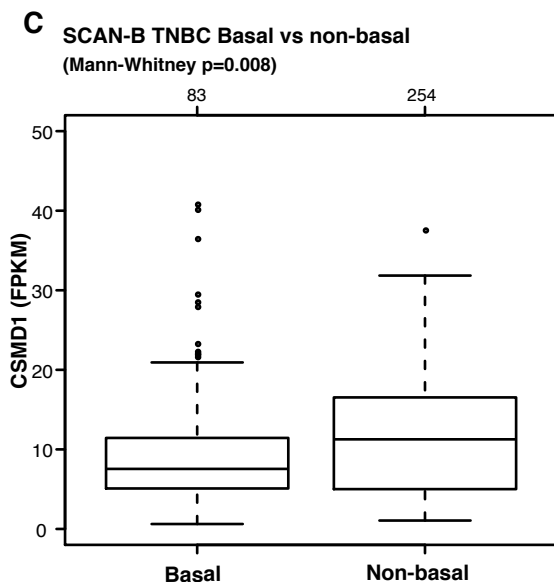

Supplement: Supplementary file 1 — Additional file 1: S.Figure 1 Expression of mRNA coding for (A) EGF, (B) TGF-α and (C) AREG in MDA-MB-231 CTRL and CSMD1 clonal cells. EGFR gene expression (FPKM) plotted against CSMD1 (FPKM) gene expression in (D) all BC patients and in (E) TNBC patients of SCAN-B cohort (F-G) Protein extracts of MDA-MB-231 BCCs were immunoprecipitated with anti-EGFR. Eluted proteins were analyzed by immunoblotting with (F) anti-phosphotyrosine (pTyr) or anti-EGFR antibody and (G) anti-phosphoserine (pSer) or anti-EGFR antibody, as indicated. (H & I) Densitometric western blot analysis of total phosphorylation tyrosine and serine residues of EGFR. Bars display mean ± SD. Mann–Whitney comparison test was used (*<0.05). (J) Binding assays with 125I-labeled EGF in CTRL and CSMD1 MDA-MB-231 BCCs. All experiments were repeated at least 3 times with bars indicating mean ± SD, grey circles correspond to independent data points for CTRL and CSMD1 groups, respectively. S.Figure 2. (A) Ubiquitinated EGFR was examined via EGFR immunoprecipitation followed by immunoblotting with anti-ubiquitin antibody in denaturing lysates. Representative blots from three independent experiments are presented in CTRL and CSMD1 MDA-MB-231 BCCs. (B) EGFR internalization kinetics using 125I-EGF in MDA-MB-231 BCCs. The amounts of internalized and surface 125I-EGF (cpm) where plotted against time upper panel, while the ratio of internalized/surface EGF against time was used to calculate the internalization rate constant ke. (C) Fractionation analysis in cytosol and membrane of CTRL and CSMD1 MDA-MB-231 BCCs upon stimulation with EGF (25 ng/mL) for 2h. Representative blots are shown. The fractions were blotted for CSMD1, EGFR, EEA1, LAMP1, β-tubulin and NA/K ATPase (D) Ratio of cytosolic to membrane EGFR was calculated. Bars display mean ± SD. S. Figure 3 Validation of the major findings in BT-20 TNBC cell line (A) Cell lysates were immunoprecipitated using antibodies against CSMD1 or corresponding IgG control foll [file 13046_2021_2042_MOESM1_ESM.zip › Supplementary Figure 6.pdf]
